# Targeted Gene Modification of *HMGR* Enhances Biosynthesis of Terpenoid and Phenylpropanoid Volatiles in Petunia and Lettuce

**DOI:** 10.3390/ijms27031522

**Published:** 2026-02-04

**Authors:** Oded Skaliter, Aviad Gura, Yarin Livneh, Raz Cohen, Elena Shklarman, Orit Edelbaum, Tania Masci, Alexander Vainstein

**Affiliations:** Institute of Plant Sciences and Genetics in Agriculture, The Robert H. Smith Faculty of Agriculture, Food and Environment, The Hebrew University of Jerusalem, Rehovot 7610001, Israel; oded.skaliter@mail.huji.ac.il (O.S.); aviadgura@gmail.com (A.G.); masci2008@gmail.com (T.M.)

**Keywords:** gene editing, lettuce, metabolic engineering, petunia, specialized metabolism, terpenoids, volatiles

## Abstract

Terpenoids constitute the largest class of plant-specialized metabolites, playing essential roles throughout the plants’ life cycle and having diverse applications for humans in nutrition, medicine, and flavor. 3-Hydroxy-3-methylglutaryl-CoA reductase (HMGR) is a rate-limiting enzyme of the mevalonate (MVA) pathway, producing sesquiterpenes, saponins, and other terpenoids. HMGR is post-translationally regulated by downstream MVA products through its N-terminal regulatory domain, limiting terpenoid production. To overcome this bottleneck, we employed a virus-based CRISPR/Cas9 system to genetically modify the N-terminal regulatory domain of *HMGR* in petunia (*Petunia × hybrida*) and lettuce (*Lactuca sativa* L.). In petunia, *HMGR1*-edited lines exhibited vigorous growth, larger flowers, and increased production of sesquiterpenes. Interestingly, they also showed enhanced production of phenylpropanoid volatiles, revealing a connection between these pathways. Transcript analysis revealed altered expression of genes involved in terpenoid biosynthesis, pyruvate metabolism, phenylpropanoid biosynthesis, and gibberellin- and auxin-related pathways, indicating enhanced carbon flux through these metabolic networks. In lettuce, *HMGR7*-edited plants displayed elevated emission of sesquiterpenes, apocarotenoids, and the phenylpropanoid benzaldehyde. Together, these results establish a transgene-free strategy to enhance the production of terpenoid and phenylpropanoid volatiles, and provide a framework for developing resilient, nutrient-enriched crops.

## 1. Introduction

**Figure 1:**
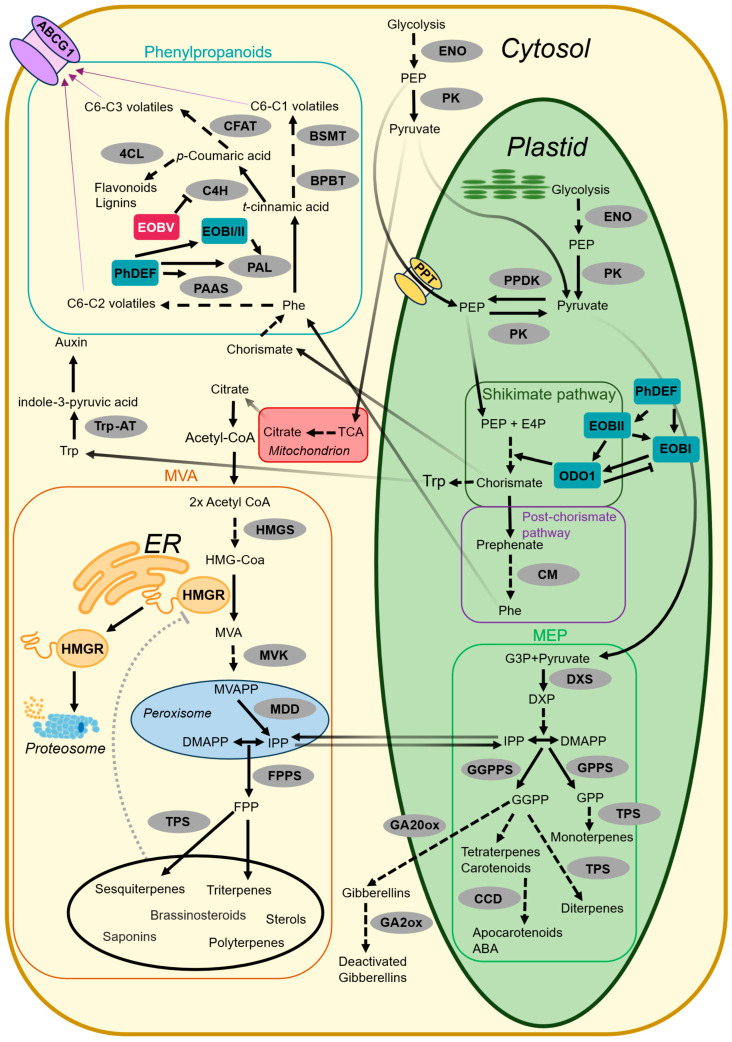
Overview of the biosynthetic pathways of terpenoids and phenylpropanoids in plants. The methylerythritol phosphate (MEP) pathway is located in the plastid, whereas the mevalonate (MVA) and phenylpropanoid pathways are primarily localized in the cytosol. The negative-feedback loop regulating hydroxy-3-methylglutaryl-CoA reductase (HMGR) (highlighted by the orange circle) is depicted. HMGR is anchored to the endoplasmic reticulum (ER) via its N-terminal transmembrane domain. Accumulation of downstream products triggers its dissociation from the ER, leading to proteasome-mediated degradation. Enzymes are shown in gray circles; positive and negative transcriptional regulators are shown in teal and red rectangles, respectively. Stacked arrows represent multiple enzymatic steps. Gray dotted arrow represents putative mechanism. Purple arrows represent volatile phenylpropanoids that are emitted via adenosine triphosphate–binding cassette (ABC) transporter (PhABCG1). Compound abbreviations: abscisic acid (ABA); acetyl coenzyme A (Acetyl-CoA); dimethylallyl diphosphate (DMAPP); 1-deoxy-D-xylulose-5-phosphate (DXP); farnesyl diphosphate (FPP); erythrose-4-phosphate (E4P); geranylgeranyl diphosphate (GGPP); geranyl diphosphate (GPP); glyceraldehyde 3-phosphate (G3P); 3-hydroxy-3-methylglutaryl-CoA (HMG-CoA); isopentenyl diphosphate (IPP); mevalonic acid (MVA); mevalonate 5-diphosphate (MVAPP); phosphoenolpyruvate (PEP); phenylalanine (Phe); tryptophan (Trp). Enzyme/transcription factor abbreviations: benzoyl-CoA:benzylalcohol/2-phenylethanol benzoyltransferase (BPBT); benzoic acid/salicylic acid carboxyl methyltransferase (BSMT); carotenoid cleavage dioxygenase (CCD); chorismate mutase (CM); cinnamate 4-hydroxylase (C4H); coniferyl alcohol acetyltransferase (CFAT); 4-coumarate-CoA ligase (4CL); deficiens (PhDEF); DXP synthase (DXS); emission of benzenoids I/II/V (EOBI/II/V); enolase (ENO); FPP synthase (FPPS); GA2ox (gibberellin 2-oxidase); GA20ox (gibberellin 20-oxidase); GGPP synthase (GGPPS); GPP synthase (GPPS); HMG-CoA synthase (HMGS); mevalonate 5-diphosphate decarboxylase (MDD); mevalonate kinase (MVK); odorant 1 (ODO1); phenylacetaldehyde synthase (PAAS); L-phenylalanine ammonia-lyase (PAL); PEP/phosphate translocator (PPT); pyruvate kinase (PK); pyruvate orthophosphate dikinase (PPDK); terpene synthase (TPS); tryptophan aminotransferase (Trp-AT). Pathway abbreviation: tricarboxylic acid (TCA).

Plants produce myriads of specialized metabolites; of these, terpenoids constitute the largest class, with over 80,000 identified compounds [1]. Owing to their biological diversity, terpenoids play essential roles in plants’ life cycle, serving as phytohormones, pigments (e.g., carotenoids), defensive compounds against pathogens and herbivores, and attractors of pollinators and seed dispersers [2]. Beyond their biological significance for plants, terpenoids are of major importance to humans; they are used in medicine (e.g., anticancer and antimalarial agents) and in the food and cosmetics industries (e.g., flavor, fragrance, and pigment compounds) [3]. In plants, terpenoid biosynthesis occurs via two pathways: the plastidic methylerythritol phosphate (MEP) pathway and the cytosolic mevalonate (MVA) pathway, both producing isopentenyl diphosphate (IPP) and its isomer dimethylallyl diphosphate (DMAPP), the building blocks of all terpenoids (Figure 1). Both pathways ultimately rely on carbon derived from pyruvate, produced via glycolysis or other sources [3]. In the first step of the MEP pathway, pyruvate is condensed with glyceraldehyde 3-phosphate (G3P) to form 1-deoxy-D-xylulose-5-phosphate (DXP), ultimately producing monoterpenes (C10), diterpenes (C20), and tetraterpenes (C40), including carotenoids, as well as the phytohormones gibberellin (GA) and abscisic acid. In the MVA pathway, pyruvate is converted to citrate in the mitochondria via the tricarboxylic acid (TCA) cycle, which is then transported to the cytosol, where it is converted to acetyl-CoA, the precursor of the MVA pathway. In addition, this pathway involves the endoplasmic reticulum (ER) and peroxisomes to generate sesquiterpenes (C15), triterpenes (C30), polyterpenes, brassinosteroids, saponins, and sterols (Figure 1) [3]. Despite their compartmental separation, the MVA and MEP pathways exchange IPP, DMAPP, and downstream intermediates, enabling metabolic crosstalk [4,5,6].

3-Hydroxy-3-methylglutaryl-CoA reductase (HMGR) is probably the first rate-limiting enzyme in the MVA pathway and is regulated by many different factors, both transcriptionally and post-translationally [3,7,8,9]. This tetrameric enzyme is responsible for the irreversible conversion of 3-hydroxy-3-methylglutaryl-CoA (HMG-CoA) to mevalonate [10]. The catalytic domain of HMGR is located in the cytosol, whereas its N-terminus is hooked to the ER membrane by a tail that, in plants, typically contains two loops [11]. Through its N-terminus, HMGR is inhibited by downstream products of the MVA pathway in a negative-feedback loop [12]. Whereas in mammals and yeast the mechanism for feedback inhibition of HMGR is well established, in plants it is still not fully elucidated [13]. Nonetheless, overexpression of a truncated form of HMGR that lacks its N-terminus resulted in elevated levels of terpenoids and sterols in plants [14,15,16,17].

Like terpenoids, phenylpropanoids—the second largest class of specialized metabolites—have a wide range of functions in plants and humans [18]. Although they are produced via distinct metabolic pathways, the biosynthesis of terpenoids and phenylpropanoids is interlinked [3,19,20,21]. For example, in tomato (*Solanum lycopersicum*), a null *chalcone isomerase* mutation affected the accumulation of both flavonoids and terpenoids in the trichomes [21]; the authors suggested that the mutation caused naringenin chalcone to accumulate, which in turn inhibited the activity of various biosynthetic and regulatory proteins. Moreover, these pathways are interlinked at the level of carbon metabolism, as phosphoenolpyruvate (PEP)—the precursor of pyruvate, a major carbon source for terpenoid biosynthesis—is also a key precursor in phenylpropanoid biosynthesis. PEP is condensed with erythrose-4-phosphate (E4P) in the shikimate pathway to generate the aromatic amino acid phenylalanine (Phe) (Figure 1) [22]. Phe, the precursor of phenylpropanoids, may undergo deamination by L-phenylalanine ammonia-lyase (PAL) to *trans*-cinnamic acid, and following a series of enzymatic reactions, lead to the production of C6-C1 (benzenoid) or C6-C3 (phenylpropene) volatiles, as well as flavonoids and lignins. Alternatively, Phe may undergo decarboxylation and oxidative deamination in a single step by phenylacetaldehyde synthase (PAAS), yielding C6-C2 (phenylpropanoid-related) volatiles [18].

Given the bioactivity of specialized metabolites, enhancing their production through metabolic engineering in both plants and microbes is a desirable biotechnological goal [17,18,23,24]. Despite the scientific successes of traditional transgenic approaches for enhancing both terpenoids and phenylpropanoids by upregulating dedicated genes [18,24], they lead to the generation of organisms classified as genetically modified (GMOs) and therefore face increased scrutiny, e.g., regulatory hurdles, high development costs, and very limited consumer acceptance. In contrast, new genomic techniques (NGTs) (also known as precision breeding), which include targeted mutagenesis (e.g., genome editing), offer a precise, transgene-free alternative for metabolic engineering. NGTs have been successfully implemented in numerous crops for the improvement of various agriculturally important and consumer-valued traits [25], and indeed, several gene-edited crops are being approved around the world [26,27], although in some countries public acceptance remains somewhat guarded [28,29].

NGTs are commonly used to generate gene knockouts, while gene upregulation remains less frequent due to its greater complexity. Recent studies have demonstrated that promoter editing can increase gene expression, but this method is largely random, requires extensive testing, and yields unpredictable results [30,31]. An alternative approach might involve knocking out repressors of the target gene, but this also requires extensive knowledge of the interplay among many regulators. Other approaches include disruption of regulatory domains involved in negative-feedback loops. For example, we and others were able to increase the transcript levels of *GDP-L-GALACTOSE PHOSPHORYLASE* (*GGP*), leading to enhanced accumulation of ascorbic acid in lettuce (*Lactuca sativa* L.). This was achieved by disrupting an upstream open reading frame found in *GGP*’s 5′UTR, known to be involved in a negative-feedback loop through which ascorbic acid regulates this enzyme’s activity [32,33]. Encouraged by these results, in this study, we explored the potential of editing the region encoding the N-terminus of *HMGR* to enhance terpenoid production in petunia (*Petunia × hybrida*) using the viral-based CRISPR/Cas9 system [33,34,35]. Flowers of *Phhmgr1*-edited petunia plants showed enhanced emission of not only sesquiterpenes but also phenylpropanoid volatiles. Moreover, the plants were more vigorous and produced larger flowers than the control plants. The observed phenotypes in the edited plants corresponded to the upregulation of multiple genes, suggesting enhanced carbon flux through the MVA, MEP, and phenylpropanoid pathways. Additionally, in lettuce, *HMGR7* editing led to an increase in the emission of terpenoids and the phenylpropanoid benzaldehyde. Taken together, we demonstrate that successful application of an NGT to remove the sequences involved in negative regulation of the enzyme HMGR leads to improved crop quality.

## 2. Results and Discussion

### 2.1. Identification of HMGRs in Petunia

**Figure 2:**
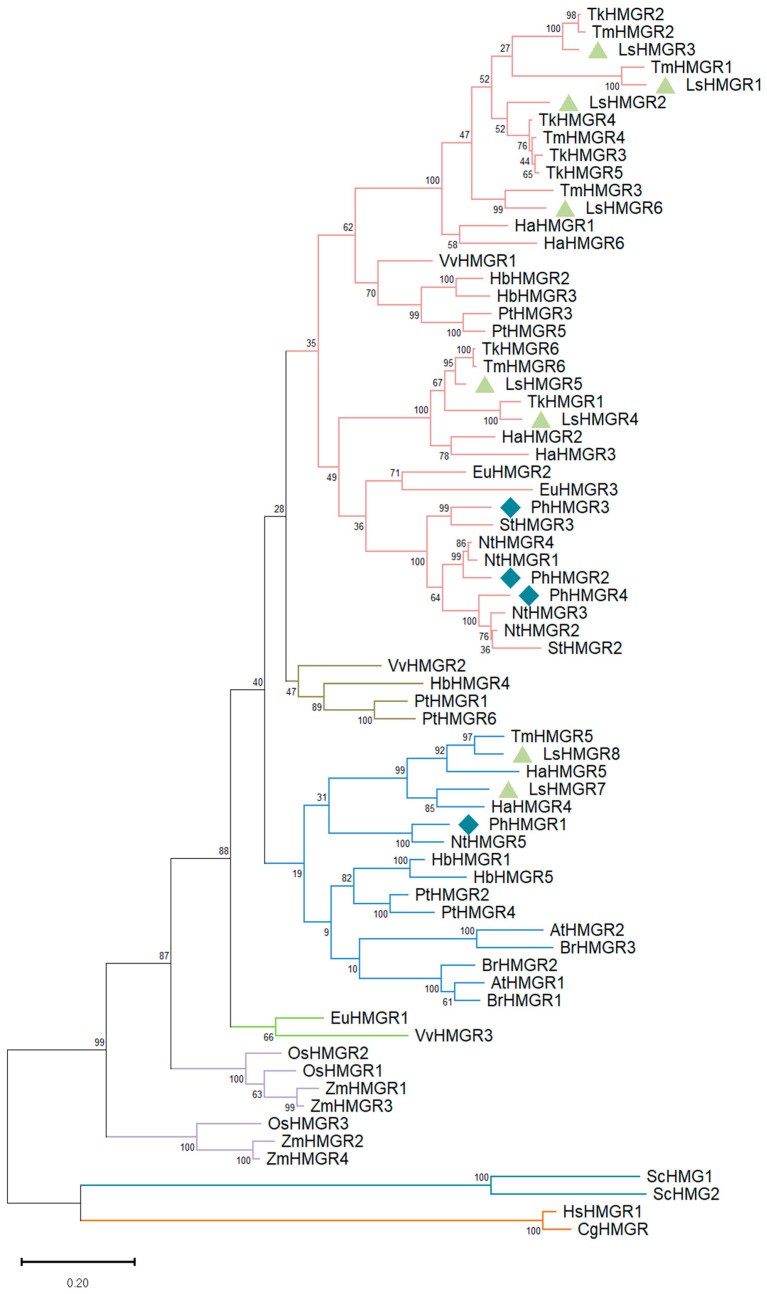
Phylogenetic analysis representing the relationships between HMGR proteins from different plant species, yeast, and mammals. Petunia and lettuce HMGR proteins are marked by diamonds and triangles, respectively. Protein sequences were aligned using ClustalW (version 2.1), followed by phylogenetic tree construction with the maximum likelihood method and 1000 bootstrap replicates in MEGA version 11. Bootstrap values are shown at branch nodes, and the scale bar represents the number of amino acid substitutions per site.

**Figure 3:**
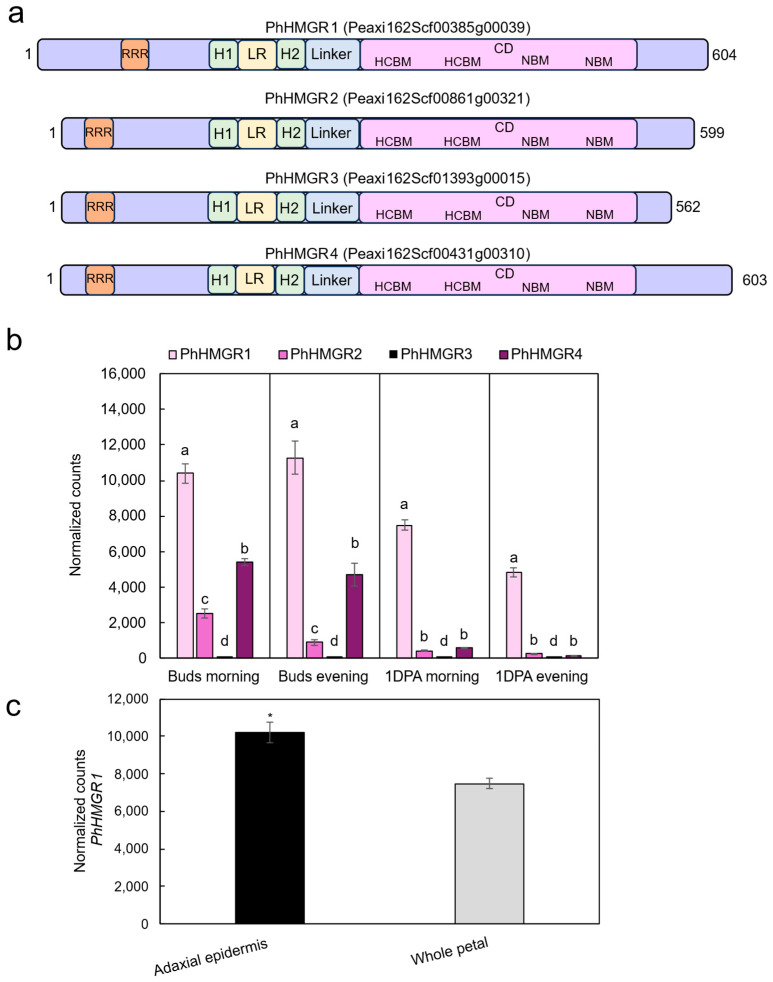
Characterization of petunia HMGRs. (**a**) Schematic representation of the domain structures of PhHMGR1, PhHMGR2, PhHMGR3, and PhHMGR4. Functional domains are indicated by colored rectangles. Numbers denote amino acid positions. CD, catalytic domain; H1/2, transmembrane domains; HCBM, HMG-CoA-binding motif; LR, lumen region; NBM, NADP(H)-binding motif; RRR, arginine-rich motif. (**b**,**c**) Developmental, temporal, and spatial expression profiles of *PhHMGR* transcripts. (**b**) Normalized counts obtained from the petunia flower transcriptome (Shor et al., 2023) [43]. Transcriptome was generated from petals of petunia cv. Mitchell harvested in the morning at 1000 h and in the evening at 1900 h from buds (4.5 cm) and opened flowers at 1 day postanthesis (1DPA). Data are means ± SEM (*n* = 3). Significance of differences for each tissue/time point was calculated by Tukey’s multiple-comparisons test following one-way analysis of variance (ANOVA). Values with different letters are significantly different at *p* ≤ 0.05. (**c**) Normalized counts of *PhHMGR1* were obtained from the transcriptome of adaxial petal epidermis vs. whole petal of petunia cv. Mitchell [34]. Data are means ± SEM (*n* = 3). Significance of differences between treatments was calculated by two-tailed unpaired Student’s *t*-test: * *p* ≤ 0.05. Standard errors are indicated by vertical lines.

The dicot genome may contain varying numbers of *HMGR* copies, ranging from two to nine [36]. For example, in *Arabidopsis thaliana*, there are two copies of *HMGR*: *AtHMGR1* and *AtHMGR2*. The former is the dominant one, as null mutants display significant pleiotropic phenotypes, such as dwarfing [37]. In contrast, *Athmgr2* mutants have no obvious phenotype, suggesting a minor or redundant role [37]. Therefore, AtHMGR1 was used to identify HMGR-encoding genes in the petunia genome by BlastP analyses performed against the genomes of *Petunia axillaris* and *Petunia inflata*, the ancestors of the garden petunia (*Petunia × hybrida*) (https://solgenomics.net/, accessed on 28 January 2026) [38]. Four putative *HMGR* homologues were identified in both ancestors: Peaxi162Scf00385g00039.1/Peinf101Scf00982g06021.1, Peaxi162Scf01393g00015.1/Peinf101Scf00393g02016.1, Peaxi162Scf00861g00321.1/Peinf101Scf00409g05014.1, and Peaxi162Scf00431g00310.1/Peinf101Scf00774g02014.1. This aligns with results from the petunia leaf transcriptome that also suggested four copies [39]. Since Peaxi162Scf00385g00039.1 showed the highest homology to AtHMGR1, it was designated PhHMGR1. Peaxi162Scf00861g00321.1 was designated PhHMGR2, and Peaxi162Scf01393g00015.1 and Peaxi162Scf00431g00310.1 were designated PhHMGR3 and PhHMGR4, respectively. Phylogenetic analysis of HMGRs from plants [40], mammals, and yeast (*Saccharomyces cerevisiae*) clustered PhHMGR1 into the same subgroup with AtHMGR1 and 2, whereas the remaining PhHMGRs clustered into a different subgroup (Figure 2). All four HMGRs had a predicted open reading frame yielding proteins with 604, 599, 562, and 603 amino acids, respectively (Figure 3a and Figure S1). Their domain structure was similar; topology prediction using DeepTMHMM (https://dtu.biolib.com/DeepTMHMM, accessed on 28 January 2026) [41] indicated that all four petunia HMGRs have two transmembrane regions in the N-terminus, as expected for plant HMGRs (Figure 3a and Figure S2). In their C-terminus, all had the two conserved HMG-CoA-binding motifs TTEGCLVA and EMPVGYVQIP, as well as the NADP(H)-binding motifs DAMGMNM and GTVGGGT (Figure 3a and Figure S1) [42]. The main difference between PhHMGR1 and the other HMGRs was the presence of an additional 24-amino acid sequence at its N-terminus, which shifts the location of the arginine-rich motif (RRR) that is important for ER retention (Figure 3a and Figure S1).

To assess the expression of the *PhHMGR*s in petunia flowers, we analyzed our previously generated petunia petal transcriptome [43]. *PhHMGR1* and *PhHMGR4* were expressed at significantly higher levels than *PhHMGR2* and *PhHMGR3* at all tested developmental and temporal stages (Figure 3b). However, *PhHMGR1* expression was the highest—approximately 2- and 14-fold higher than *PhHMGR4* in buds and mature flowers 1 day postanthesis (1DPA), respectively. *PhHMGR3* was expressed at background levels in buds and mature flowers, similar to its expression levels in petunia leaves [39]. This suggests that *PhHMGR3* has a limited role in terpenoid biosynthesis and/or that its function is restricted to specific physiological/stress conditions, possibly like AtHMGR2 [37]. The expression of *PhHMGR1* and *PhHMGR4* was highest in buds, which have been shown to accumulate high levels of terpenoids compared to mature flowers [44]. *PhHMGR4* expression greatly declined in mature flowers, whereas *PhHMGR1* maintained high expression levels. *PhHMGR1* was also highly expressed in leaves, suggesting that it might play a role in that tissue as well [39]. Interestingly, at 1DPA, *PhHMGR1* exhibited a diel expression pattern—higher in the morning than in the evening (Figure S3). Moreover, analysis of the petunia adaxial petal RNA-sequencing dataset [34] showed that *PhHMGR1* expression is enriched in the adaxial epidermis—the main site of volatile production (Figure 3c) [45]. Given that PhHMGR1 was most similar to AtHMGR1, had the highest expression in flowers, and was enriched in the adaxial epidermis, we selected it as a target for genome editing with the aim of enhancing terpenoid production in petunia flowers.

### 2.2. Gene-Editing of PhHMGR1 Using a Virus-Based CRISPR/Cas9 System

**Figure 4:**
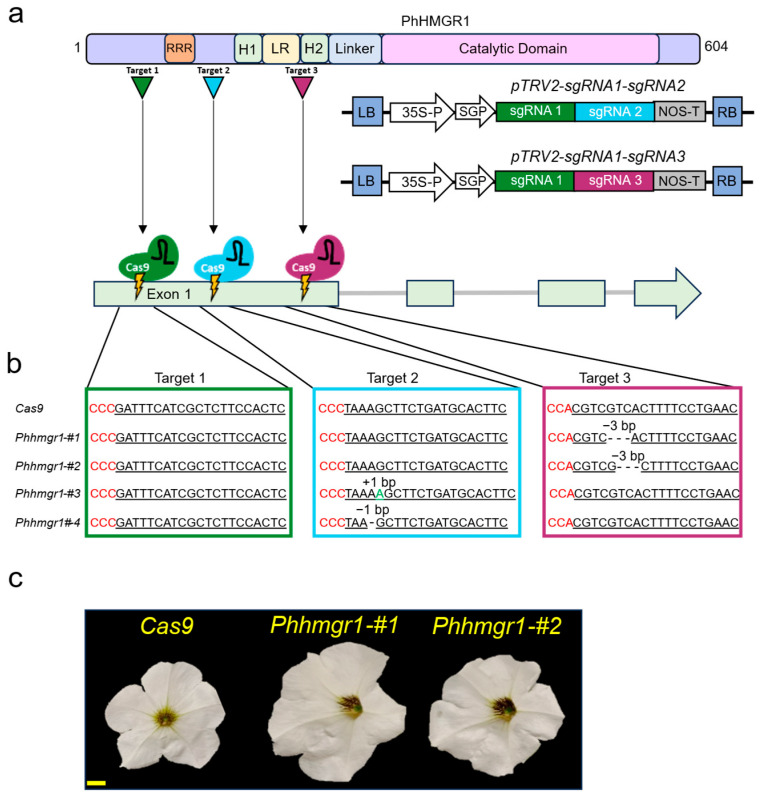
Generation of *PhHMGR1*-edited lines using a virus-based Cas9 system. (**a**) Top: schematic representation of the domain structure of PhHMGR1 protein. Triangles mark the locations of the areas targeted by the virus-based Cas9 system. Numbers denote amino acid positions. H1/2, transmembrane domains; LR, lumen region; RRR, arginine-rich motif. Middle: schematic representation of the T-DNA carrying tobacco rattle virus RNA2 (pTRV2) vectors used for gene editing. LB, left border; 35S-P, cauliflower mosaic virus 35S promoter; SGP, subgenomic promoter; sgRNA, single guide RNA; NOS-T, nopaline synthase terminator; RB, right border; Cas9, human codon-optimized Cas9 gene. Bottom: schematic representation of the *PhHMGR1* gene structure and the genomic locations targeted by sgRNA1, 2, and 3. (**b**) Targeted genomic sequences of *Phhmgr1* in control and edited lines. Red nucleotides, protospacer adjacent motif (PAM); green nucleotide, nucleotide insertion; underlined nucleotides, spacer sequence. (**c**) Representative petunia cv. Mitchell flowers 1DPA from control *Cas9* and *Phhmgr1* lines #1 and #2. Bar = 1 cm.

In an attempt to enhance terpenoid biosynthesis in petunia flowers, we targeted the N-terminal domain of *PhHMGR1* with the previously developed virus-based CRISPR/Cas9 system [34]. Three spacers were designed using the CRISPOR tool (version 5.2) [46]. The single-guide RNAs (sgRNAs) were cloned into two tobacco rattle virus vectors (pTRV2): pTRV2-sgRNA1-sgRNA2 and pTRV2-sgRNA1-sgRNA3 (Figure 4a). sgRNA1 and sgRNA2 targeted the region upstream and downstream of the sequence encoding the RRR motif, respectively [47]; sgRNA3 targeted the lumen region and transmembrane region 2 (Figure 4a). *Cas9*-expressing explants [34] were inoculated with agrobacteria harboring either pTRV2-sgRNA1-sgRNA2 or pTRV2-sgRNA1-sgRNA3. Two lines were generated from the plantlets inoculated with the latter, harboring 3-bp deletions in the sgRNA3 target site. Deletion of nucleotides 346–348 in *Phhmgr1*-#1 led to the loss of valine, and deletion of nucleotides 347–349 in *Phhmgr1*-#2 led to loss of valine and substitution of threonine by alanine (Figure 4b). From explants inoculated with the pTRV2-sgRNA1-sgRNA2, two lines were generated harboring mutations in the site targeted by sgRNA2, leading to premature stop codons due to frameshift (FS) mutations: an adenine insertion in line *Phhmgr1*-#3 and an adenine deletion in line *Phhmgr1*-#4 (Figure 4b). The frameshift mutant lines were not further characterized, as the T2 homozygous plants exhibited a severe dwarf phenotype, as also reported for *Arabidopsis* mutants with loss of function of *Athmgr1* (Figure S4) [37].

### 2.3. Phhmgr1 Mutation Boosts Emission and Production of Terpenoids and Phenylpropanoids in Petunia Flowers

**Figure 5:**
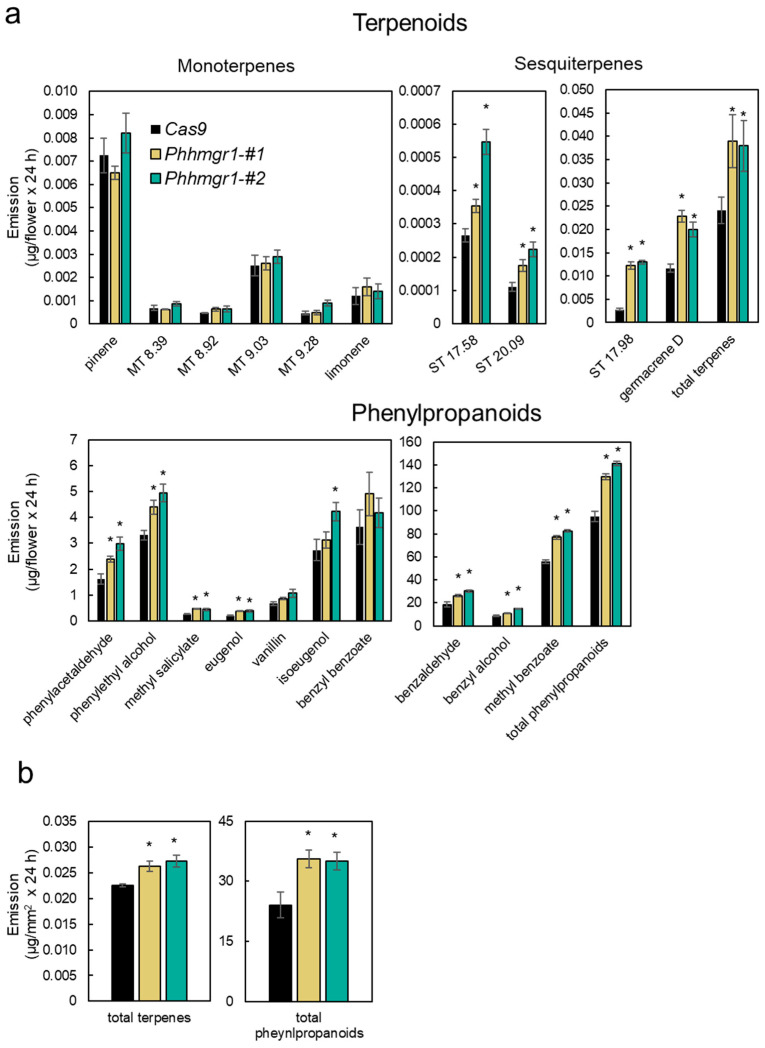
Flowers of the gene-edited *Phhmgr1-#1* and *Phhmgr1-#2* lines exhibit increased emission of terpenoids and phenylpropanoids. (**a**) Dynamic headspace analyses of *Phhmgr1*-edited lines and *Cas9* control were performed for 24 h on flowers at 1DPA followed by GC–MS analyses. Data are means ± SEM (*n* = 4–6). MT, monoterpenes; ST, sesquiterpenes. Numbers next to compounds denote their GC retention time. (**b**) Localized headspace analyses of *Phhmgr1*-edited lines and *Cas9* were performed on flowers harvested 1DPA for 24 h followed by GC–MS analyses. Data are means ± SEM (*n* = 3). Significance of differences was calculated using Dunnett’s test (* *p* ≤ 0.05) with Cas9 as the control following one-way ANOVA. Standard errors are indicated by vertical lines.

Both edited lines, *Phhmgr1*-#1 and *Phhmgr1*-#2, from the T2 generation had bigger seeds, thicker stems and leaves, and longer internodes; were more vigorous; and had larger flowers than *Cas9* control plants (Figure 4c and Figure S5a–c). Similar phenotypes of increased biomass have been reported in transgenic potato (*Solanum tuberosum*) plants overexpressing *HMGR* [48], attributed to increased levels of sterols, which are derived from the MVA pathway and play key roles in plant development and growth by affecting phytohormones and cellulose biosynthesis [49,50]. To evaluate the effect of mutations in *PhHMGR1* on terpenoid emission, we conducted dynamic headspace analysis on flowers of the CRISPR-edited lines. Flowers of both *Phhmgr1* lines demonstrated a significant increase in total terpenoid levels (Figure 5a). All detected sesquiterpenes significantly increased, including a ca. two-fold increase in germacrene D, the dominant terpenoid in the petunia volatilome [51]. In contrast, emission levels of detected monoterpenes were unaltered in *Phhmgr1*-edited lines relative to control *Cas9*. Because petunia flowers produce mostly phenylpropanoid volatiles [51], and phenylpropanoid and terpenoid biosynthesis have been shown to be linked [3,20,21,52], we also tested emission levels of these compounds in flowers of *Phhmgr1*-edited lines. Similar to terpenoids, total phenylpropanoid emission was upregulated in *Phhmgr1* flowers compared to control *Cas9*. All phenylpropanoid volatiles showed a significant increase in emission levels, except for isoeugenol, whose increase was only significant in *Phhmgr1*-#2, and benzyl benzoate and vanillin, which were unaltered in the edited lines (Figure 5a). To evaluate whether the differences in emission levels of terpenoids and phenylpropanoids can be attributed to the larger diameter of *Phhmgr1* flowers, we performed localized headspace analysis [45], sampling volatiles that are emitted from a given surface area of the petal. Similar to the results of the dynamic headspace analysis, localized analysis revealed a significant increase in emissions of both terpenoids and phenylpropanoids in flowers of the *Phhmgr1* lines (Figure 5b). These results indicate that in-frame mutations introduced into the N-terminal coding region of *PhHMGR1* result in enhanced emission of not only terpenoids but also phenylpropanoid volatiles in petunia flowers, revealing a metabolic interconnection between these two pathways in petunia. To assess whether the mutations in *Phhmgr1* also affect the accumulation of volatiles, internal pools were analyzed in flowers of the *Phhmgr1*-edited lines and compared to control *Cas9* plants. In line with the dynamic and localized headspace results, *Phhmgr1* lines exhibited a significant increase in the internal pools of the terpenoid germacrene D and the phenylpropanoids eugenol, methyl benzoate, phenylethyl alcohol, and benzyl alcohol (Figure S6). Conversely, the isoeugenol pool was reduced only in *Phhmgr1*-#1 and benzyl benzoate was reduced only in *Phhmgr1*-#2. Internal pool levels of other identified volatiles were unaltered. The uncoupling between headspace and internal pools of some of the compounds highlights the complexity of the machinery controlling volatile biosynthesis and inter- and intracellular transport, previously described in rose and petunia [20,34,53,54]. Despite this specific discrepancy, the headspace and internal pool analyses indicated that the *Phhmgr1* mutation affects not only the emission of terpenoid and phenylpropanoid volatiles but also their accumulation. However, the affected terpenoids derived exclusively from the MVA pathway, with no significant impact on MEP-derived volatile terpenoids despite these pathways’ interconnection. In contrast, the effects on phenylpropanoids were broader, with increases observed across all phenylpropanoid branches (C6-C1, C6-C2, and C6-C3).

### 2.4. Expression of Genes Encoding Key Enzymes in Terpenoid and Phenylpropanoid Biosynthesis

**Figure 6:**
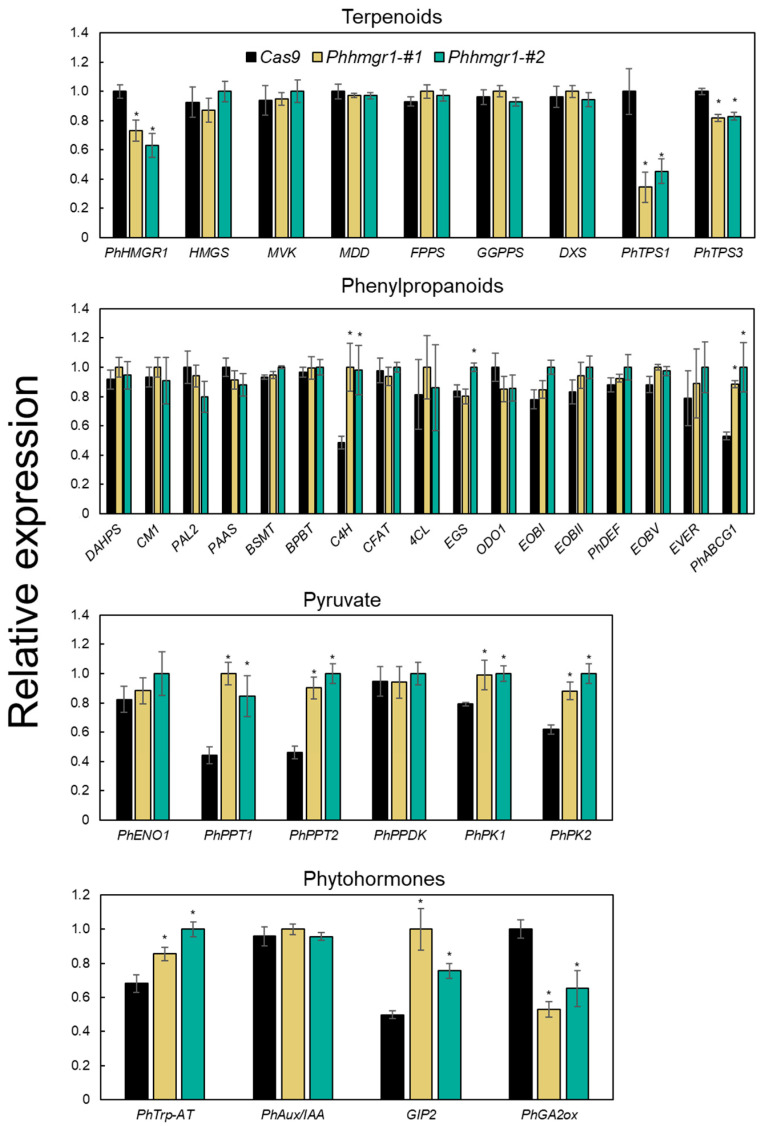
Gene-edited *Phhmgr1-#1* and *Phhmgr1-#2* lines exhibit altered expression of genes involved in terpenoid, phenylpropanoid, pyruvate, and phytohormones production. Reverse transcription quantitative PCR analyses were performed on RNA extracted from flowers at 1 day postanthesis (1DPA) of *Phhmgr1-#1* and *Phhmgr1-#2* and *Cas9* control lines at 1800 h. Data are means ± SEM (*n* = 3–4). Data were normalized to the geometric mean of *Ubiquitin* and *EF1α*. Significance of differences was calculated using Dunnett’s test (* *p* ≤ 0.05) with Cas9 as the control following one-way ANOVA. Standard errors are indicated by vertical lines. Enzyme/transcription factor abbreviations: adenosine triphosphate–binding cassette (abc) transporter (PhABCG1); auxin/indole-3-acetic acid (PhAux/IAA); benzoyl-CoA:benzylalcohol/2-phenylethanol benzoyltransferase (BPBT); benzoic acid/salicylic acid carboxyl methyltransferase (BSMT); chorismate mutase 1 (CM1); cinnamate 4-hydroxylase (C4H); coniferyl alcohol acetyltransferase (CFAT); 4-coumarate-CoA ligase (4CL); deficiens (PhDEF); 3-deoxy-D-arabinoheptulosonate 7-phosphate synthase (DAHPS); 1-deoxy-ᴅ-xylulose-5-phosphate synthase (DXS); emission of benzenoids I/II/V (EOBI/II/V); enolase (PhENO1); eugenol synthase (EGS); epidermis volatile emission regulator (EVER); farnesyl diphosphate synthase (FPPS); gibberellin-induced protein 2 (GIP2); gibberellin 2-oxidase (GA2ox); gibberellin 20-oxidase (GA20ox); geranylgeranyl diphosphate synthase (GGPPS); hydroxy-3-methylglutaryl-coa reductase 1 (PhHMGR1); 3-hydroxy-3-methylglutaryl-CoA synthase (HMGS); mevalonate 5-diphosphate decarboxylase (MDD); mevalonate kinase (MVK); odorant 1 (ODO1); phenylacetaldehyde synthase (PAAS); phenylalanine ammonia-lyase 2 (PAL2); phosphoenolpyruvate/phosphate translocator 1/2 (PhPPT1/2); pyruvate kinase 1/2 (PhPK1/2); pyruvate orthophosphate dikinase (PhPPDK); terpene synthase 1/3 (PhTPS1/3); tryptophan aminotransferase (PhTrp-AT).

To detail the effect of the *Phhmgr1* mutation on the expression of key genes involved in terpenoid biosynthesis, their transcript levels were analyzed in *Phhmgr1* lines by reverse transcription quantitative PCR (RT-qPCR). Despite the significant increase in terpenoid production, transcript levels of *PhHMGR1* were reduced in flowers of both *Phhmgr1* lines (Figure 6). This suggests that gene editing of the N-terminus of *PhHMGR1*—aimed at releasing negative feedback at the protein level—affects the steady-state level of its mRNA. This is consistent with previous reports showing that HMGRs are heavily regulated at both the post-transcriptional and translational levels [7,8,9]. As terpenoid-pathway genes have not been detailed in petunia, we screened the petunia flower transcriptome [43] for putative *HYDROXYMETHYLGLUTARY*L-*CoA SYNTHASE* (*HMGS*), *MEVALONATE 5-DIPHOSPHATE DECARBOXYLASE* (*MDD*), *MEVALONATE KINASE* (*MVK*), *FARNESYL DIPHOSPHATE SYNTHASE* (*FPPS*), and *GERANYLGERANYL DIPHOSPHATE SYNTHASE* (*GGPPS*). We found only one putative *MDD*, *MVK*, and *FPPS* (Figure S7). By comparison, there were two and four putative *HMGS*s and *GGPPS*s, respectively, and therefore we selected the candidates with the highest expression level in mature flowers (1DPA). RT-qPCR analyses revealed that the expression levels of all tested genes were unaltered, meaning that increased production of terpenoids is not due to changes in the expression of these genes (Figure 6). To test the expression of *TERPENE SYNTHASE* (*TPS*), which encodes dedicated enzymes in terpenoid production, we screened the transcriptomic database for *TPS* genes that are highly expressed in petals [43]. Among the 10 candidates, 2 stood out: Peaxi162Scf00074g00143 and Peaxi162Scf00190g01521 (Figure S7), which were previously identified by Boachon et al. [44] as *PhTPS1* and *PhTPS3*, respectively. *PhTPS1* was shown to produce a dozen sesquiterpenes, including germacrene D [44], which was increased in both *Phhmgr1* lines (Figure 5a). Expression analysis of *PhTPS1* and *PhTPS3* in *Phhmgr1* mutants revealed decreases in both lines (Figure 6). This result suggests that increased levels of terpenoids in edited lines trigger a negative-feedback loop on *PhTPS1/3*. A similar phenomenon has been observed in *Artemisia annua*, where spraying leaves with the sesquiterpenoid artemisinic acid led to suppression of its dedicated *TPS*, *AMORPHA-4*, *11-DIENE SYNTHASE* [55]. Moreover, a decrease in the transcript levels of phenylpropanoid-dedicated enzymes was also observed in petunia flowers hyperaccumulating volatiles, perhaps to prevent autotoxicity, which could compromise cellular compartment integrity [53].

As emission of phenylpropanoids also increased in *Phhmgr1* lines, we analyzed transcript levels of pathway-related genes, as well as aromatic amino acid biosynthesis. None of the positive transcriptional regulators tested, i.e., *EMISSION OF BENZENOIDS I* and *II* (*EOBI/II*) and *ODORANT1* (*ODO1*), showed any difference in expression in the *Phhmgr1* lines compared to *Cas9* (Figure 6). Accordingly, transcripts of *PhDEF*, shown to control those regulators, as well as of *CHORISMATE MUTASE1* (*CM1*) and *3-DEOXY-D-ARABINO-HEPTULOSONATE-7-PHOSPHATE SYNTHASE* (*DAHPS*), were also unaltered in those lines [56], and the levels of the tested negative regulators—*EMISSION OF BENZENOIDS V* (*EOBV*) and *EPIDERMIS VOLATILE EMISSION REGULATOR* (*EVER*)—were also comparable to those in *Cas9* control flowers. In line with the stark emission increase in volatile phenylpropanoids in *Phhmgr1* lines, mRNA levels of the volatile transporter *ATP-BINDING CASSETTE SUBFAMILY G MEMBER 1* (*PhABCG1*) was significantly elevated (Figure 6). Furthermore, flowers of *Phhmgr1* lines exhibited elevated levels of transcripts encoding *cinnamate 4-hydroxylase* (*C4H*), controlling carbon flux toward phenylpropene biosynthesis [57], and *eugenol synthase* (*EGS*), the dedicated enzyme producing eugenol, aligning with the enhanced emission of eugenol in *Phhmgr1* lines. Expression levels of other tested genes encoding biosynthetic enzymes of the aromatic amino acid and phenylpropanoid pathways were similar to those in *Cas9*. These results indicate that the observed increase in volatile phenylpropanoid levels is not associated with changes in the expression of the known pathway regulators examined here, and is likely mediated through indirect mechanisms, such as altered metabolic flux or precursor availability. This notion may explain the increased expression of *PhABCG1*, *C4H*, and *EGS*, as increased carbon flux has previously been shown to affect phenylpropanoid-related genes in *Arabidopsis* and grape (*Vitis vinifera*) [58,59].

### 2.5. Expression of Genes Encoding Key Enzymes in Pyruvate Metabolism Is Altered in Phhmgr1 Mutants

Because pyruvate represents a central metabolic link between terpenoid and phenylpropanoid biosynthesis, we detailed the expression of genes encoding pyruvate kinases (*PK*s), which catalyze the final step of glycolysis, converting PEP to pyruvate. Currently, there are no functionally characterized PKs in petunia, and among the 10 putative *PK*s identified in the transcriptomic database [43], Peaxi162Scf00110g01213 (dubbed *PhPK1*) and Peaxi162Scf00329g00422 (*PhPK2*) showed the highest expression levels (Figure S7). Protein domain analysis using InterPro (version 107.0) [60] showed that both have conserved PK domains and probably reside in the cytoplasm, as they have no predicted chloroplast signal (https://services.healthtech.dtu.dk/services/TargetP-2.0/, accessed on 28 January 2026). Although pyruvate may be supplied via either cytosolic or plastidial glycolysis, it probably relies more on the former, as suppressing pyruvate production via the cytosolic route in petunia flowers resulted in a significant reduction in anthocyanins, which are products of the phenylpropanoid pathway [61]. In both *Phhmgr1* lines, RT-qPCR analysis showed significantly higher expression of both *PhPK*s compared to *Cas9* control (Figure 6). To evaluate the expression of additional key genes involved in pyruvate metabolism and transport, we examined *PhENOLASE1* (*PhENO1*), which generates PEP via glycolysis; *PhPHOSPHOENOLPYRUVATE/PHOSPHATE TRANSLOCATOR1* (*PhPPT1*) and *PhPPT2*, which import PEP from the cytosol into plastids; and *PhPYRUVATE ORTHOPHOSPHATE DIKINASE* (*PhPPDK*), which converts pyruvate to PEP. These genes were previously identified and characterized in petunia cv. Ultra [61]. In flowers of both *Phhmgr1* lines, transcript levels of *PhENO1* and *PhPPDK* were unaltered in comparison to control *Cas9* flowers (Figure 6). Conversely, levels of both *PhPPT* isoforms were ca. twofold higher in *Phhmgr1* lines. Both *PhPPT1* and *PhPPT2* are expressed in petunia flowers; the former mainly in the corolla and the latter mainly in the tube—the main site of terpenoid biosynthesis [44,61]. The increased expression of both *PhPPT* isoforms suggests enhanced PEP flux into floral plastids in both the tube and the corolla, together with enhanced pyruvate production driven by increased *PK* levels, which may indicate enhanced available carbon flux for both terpenoid and phenylpropanoid pathways. Similarly, studies in petunia and rose have shown that increasing carbon flux into these pathways enhances volatile emission [20,62,63]. Nevertheless, direct measurements of carbon flux, such as carbon-label feeding experiments in *Phhmgr1* lines, will be necessary to fully validate this conclusion.

### 2.6. Expression of Genes Encoding Key Enzymes in Biosynthesis and Signal Transduction of Hormones Is Altered in Phhmgr1 Mutants

*Phhmgr1*-edited lines displayed a vigorous phenotype with bigger seeds, thicker stems and leaves, longer internodes, and larger flowers as compared to *Cas9* control plants (Figure 4c and Figure S5a–c). This phenotype is similar to that of petunia plants with enhanced auxin transport [64]. The main active auxin, indole-3-acetic acid (IAA), is produced from the aromatic amino acid tryptophan (Trp) [65] via the shikimate pathway. Tryptophan aminotransferase (Trp-AT) converts TRP to indole-3-pyruvic acid (IPA), the precursor of IAA, and its expression is increased in response to IAA treatment [66]. A petunia homologue of Trp-AT was recently identified and characterized [67]. Transcript levels of auxin/indole-3-acetic acid (Aux/IAA) proteins are another indicator of auxin activity, as high auxin levels trigger their degradation while simultaneously upregulating their transcript levels to maintain homeostasis [68]. Auxins have extensive crosstalk and overlapping activities with GAs; the latter also promote organ growth, including petal size [69,70,71]. GAs are formed via the MEP pathway [72] by multiple enzymes. Gibberellin 2-oxidases (GA2ox), which are responsible for deactivation of GAs, serve as indicators of GA status: high GA levels induce their expression, reflecting feed-forward regulation for maintenance of GA homeostasis [73,74]. In addition, GA-induced protein 2 (GIP2) has also been used by us and others as a marker of elevated GA levels in petunia [34,75,76]. To test if the phenotypes observed in *Phhmgr1* lines may be attributed to altered levels of auxin and/or GA, we tested the expression of *PhTrp-AT*, *GIP2*, and putative *Aux/IAA* and *GA2ox*. As the latter two are not functionally characterized in petunia, we screened the petunia transcriptome and selected Peaxi162Scf01416g00118 (*PhAux/IAA*) and Peaxi162Scf00111g00922 (*PhGA2ox*) for analysis due to their high expression in mature flowers (Figure S7). Surprisingly, RT-qPCR analysis showed that the expressions of *GIP2* and *PhGA2ox* changed in opposite directions, with the former being upregulated and the latter downregulated in *Phhmgr1* lines compared to *Cas9* controls (Figure 6). At first, this result seems counterintuitive, because GA is generally expected to increase the expression of both genes. However, some GA2ox isoforms do not follow the canonical GA-induced homeostatic pattern. For example, in *Solanum pennellii*, GA_3_ treatment leads to a decrease in the expression of specific *GA2ox* transcripts [77]. Moreover, *GA2ox* expression has been reported to be repressed by auxin [78], and indeed, the expression of *PhTrp-AT* was upregulated in *Phhmgr1* lines, indicating high auxin levels in flowers of those lines (Figure 6). *PhAux/IAA* transcript levels were unaltered in *Phhmgr1*, probably because different *Aux/IAA* genes respond differently to auxin [79]. Taken together, these results imply that both auxin and GA biosynthesis and signaling are upregulated in *Phhmgr1* lines, possibly explaining their observed vigorous phenotype. The increase in GA in *Phhmgr1* lines is consistent with a previous study showing that overexpression of *HMGR* in *Populus trichocarpa* results in a significant increase in GA_3_ levels [80]. Because GAs are produced via the MEP pathway, we tested the expression levels of *1-DEOXY-D-XYLULOSE 5-PHOSPHATE SYNTHASE* (*DXS*), catalyzing the pathway’s rate-limiting step. Of the two putative *DXS* candidates in the petal transcriptome, Peaxi162Scf00048g02226 was chosen for further analysis, as it showed higher expression and homology to *DXS2* from tomato (Figure S7) [81]. However, its expression was unaltered in *Phhmgr1* lines. Although the MVA and MEP pathways have been reported to be interconnected at the transcriptional level [82], the lack of effect on *DXS* expression supports the notion that the increase in GA levels is probably not due to transcriptional upregulation of genes encoding dedicated enzymes of the MEP pathway. Despite their compartmental separation, the MVA and MEP pathways are known to exhibit metabolic crosstalk through the exchange of IPP, DMAPP, and other downstream products. The extent and direction of this crosstalk is dependent on the species, organ, and developmental stage [3]. This intercompartmental movement of metabolites between MVA and MEP pathways may facilitate the increase in carbon levels in the plastids, leading to enhanced production of GA in *Phhmgr1* lines. The increase in carbon flux, as may be assumed by the elevated expression of *PhPK*s and *PhPPT*s (Figure 6), may also aid in overcoming the negative effect of auxin and GA on volatile production [67,76] in *Phhmgr1* lines.

Overall, targeted editing of specific sequences in *Phhmgr1* was shown to effectively enhance the production and emission of terpenoids and phenylpropanoids in petunia flowers, further demonstrating the applicability of NGTs in metabolic engineering. The molecular and phenotypic data presented here indicate that the induced in-frame mutations in *PhHMGR1* generated by the virus-based CRISPR system alleviated post-translational negative feedback regulation of the HMGR1 enzyme; however, further research at the protein level and on enzymatic activity is required to fully confirm this conclusion. Beyond its scientific value, such as revealing an additional layer of interconnection between multiple metabolic pathways, this work has clear implications for the molecular breeding of agriculturally important plants. Floral volatiles play a role in plant defense, human health, and flavor, and are crucial for successful animal-mediated pollination, which is responsible for approximately one-third of global crop production by volume [83]. Although floral scent has often been overlooked in breeding programs, it is of particular importance for ornamental crops due to its consumer value [84]. In addition, the vigorous phenotypes generated in this study, characterized by larger flowers, further enhance ornamental value, as the industry is continually seeking cultivars with novel and appealing traits. Collectively, this work opens new avenues for improving both the esthetic and sensory qualities of crops.

### 2.7. Gene Editing of HMGR7 in Lettuce Leads to an Increase in Terpenoids and Phenylpropanoids

**Figure 7:**
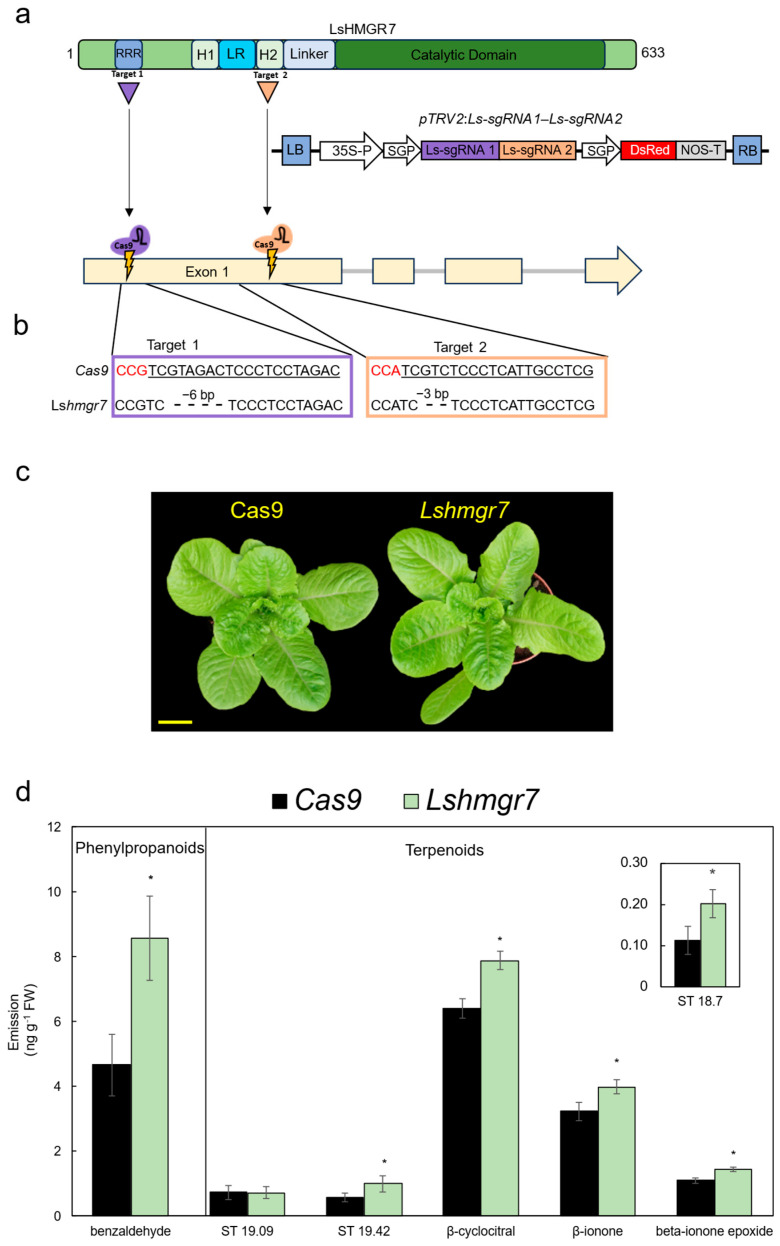
Gene editing of *LsHMGR7* results in enhanced emission of terpenoids and phenylpropanoids from lettuce leaves. (**a**) Top: schematic representation of the domain structure of LsHMGR7. Triangles mark the locations of the areas targeted by the virus-based Cas9 system. Numbers denote amino acid positions. H1/2, transmembrane domains; LR, lumen region; RRR, arginine-rich motif. Middle: schematic representation of the T-DNA carrying tobacco rattle virus RNA2 (pTRV2) vector used for gene editing. LB, left border; 35S-P, cauliflower mosaic virus 35S promoter; SGP, subgenomic promoter; Ls-sgRNA, single guide RNA; DsRed, discosoma red fluorescent protein; NOS-T, nopaline synthase terminator; RB, right border; Cas9, human codon-optimized Cas9 gene. Bottom: schematic representation of the *LsHMGR7* gene structure and the genomic locations targeted by the sgRNAs. (**b**) Targeted genomic sequences of *Lshmgr7*-edited line. Red nucleotides, protospacer adjacent motif (PAM); underlined nucleotides, spacer sequence. (**c**) Representative 1-month-old *Cas9* (control) and *Lshmgr7* lettuce plants. Bar = 5 cm. (**d**) Dynamic headspace analyses of leaves harvested from 1-month-old *Cas9* (control) and *Lshmgr7* plants were performed for 24 h followed by GC–MS analyses. ST, sesquiterpenes. Data are means ± SEM (*n* = 3). Significance of differences between treatments was calculated by two-tailed unpaired Student’s *t*-test: * *p* ≤ 0.05. Standard errors are indicated by vertical lines.

The Asteraceae is one of the largest plant families, with over 25,000 species. It includes many economically important crops grown for food and oil, among other uses, such as sunflower (*Helianthus annuus*) and lettuce (*Lactuca sativa*). Lettuce is a widely consumed leafy vegetable, valued for its crisp texture and low caloric content; however, its overall nutritional value is relatively limited. Given that terpenoids produced via the MVA/MEP pathways have nutritional value, possess antioxidant activity, and also contribute to flavor, we tested whether terpenoid levels could also be enhanced in lettuce by genetically modifying *HMGR*. To identify putative HMGRs in lettuce, we performed BlastP analysis using AtHMGR1 against the lettuce RefSeq database (https://www.ncbi.nlm.nih.gov/, accessed on 28 January 2026); this revealed eight candidates, similar to the number of HMGR copies reported in a close Asteraceae relative by Du et al. [40]. In the phylogenetic analysis, LsHMGR7 and LsHMGR8 clustered into the same group as AtHMGR1 and PhHMGR1 (Figure 2), whereas the remaining six candidates clustered together in the other major dicot subgroup. To detail which of the candidates is highly expressed in the edible leaves, the expression profile of *LsHMGR*s was analyzed in the lettuce transcriptome generated from above-ground tissues [85]. The expression levels of *LsHMGR7*, *LsHMGR3*, and *LsHMGR4* were significantly higher than those of the other five *LsHMGR* genes, which were expressed at near-background levels (Figure S8a). Among these top three, *LsHMGR7* expression was ca. two and three times higher than *LsHMGR3* and *LsHMGR4*, respectively (Figure S8a). Based on these results, *LsHMGR7* was selected for gene editing using the viral-based CRISPR/Cas9 system developed for lettuce [33,35]. The domain structure of LsHMGR7 was similar to that of PhHMGR1, with two transmembrane regions and the crucial RRR motif in its N-terminus (Figure 7a and Figure S8b). Two spacers were designed and cloned into the pTRV2 vector (which also harbored DsRed as a marker gene for virus spread): one targeting the RRR domain and the second targeting the transmembrane region 2 (Figure 7a). To generate *Lshmgr7*-edited lettuce plants, cotyledons of Cas9-expressing lettuce cv. Noga [33] were inoculated with pTRV2:Ls-sgRNA1-Ls-sgRNA2-DsRed. Plantlets regenerated from *DsRed*-expressing explants were selected for further analyses (Figure S8c). Generated gene-edited lines harboring FS mutations leading to premature stop codons: *Lshmgr7-FS1* and *Lshmgr7-FS2*, with 11-bp and 287-bp deletions (Figure S8d), respectively, did not develop properly—they were much smaller than control plants and failed to produce seeds (Figure S8e). A similar phenotype has been observed in mutants of petunia (Figure S4) and *Arabidopsis* [37]. In contrast, line *Lshmgr7*, which had two in-frame mutations—a 6-bp deletion in target 1 (resulting in the loss of two arginines in the RRR domain) and a 3-bp deletion in target 2 (resulting in loss of valine in transmembrane region 2)—developed normally and was phenotypically comparable to control *Cas9* plants (Figure 7b,c). To evaluate the effect of the genetic modification of *LsHMGR7* on terpenoid levels, they were examined in leaves of the *Lshmgr7*-edited line and compared to control *Cas9* leaves by gas chromatography–mass spectrometry (GC–MS). Six terpenoids were detected: three sesquiterpenes and the apocarotenoids β-cyclocitral, β-ionone, and epoxy-β-ionone (Figure 7d). Whereas sesquiterpenes are produced via the MVA pathway, apocarotenoids are derived from cleavage of tetraterpenes, such as β-carotene and zeaxanthin, produced via MEP [86,87]. Of the six identified compounds, emission levels of all except the sesquiterpene with retention time 19.09 were significantly elevated in *Lshmgr7* relative to the control *Cas9* plants (Figure 7d). The increase in sesquiterpenes may be attributed to enhanced flux into the MVA pathway due to the modification of HMGR7. The crosstalk between the MVA and MEP pathways, as was also observed in *PhHMGR1* lines, may explain the increase in apocarotenoid levels. In accordance, we previously showed that *lycopene ε-cyclase* (*LCY*-ε)-knockout lettuce plants with enhanced metabolic flux toward β-branch carotenoids exhibited ca. twofold higher emission levels of β-ionone [33].

Lettuce also produces volatile phenylpropanoids [88]. Indeed, in our GC–MS experiments, we detected emission of the C6-C1 compound benzaldehyde from lettuce leaves (Figure 7d). Similar to the headspace results observed in petunia, benzaldehyde levels were approximately twofold higher in *Lshmgr7* plants than in the *Cas9* control (Figure 7d). This indicates that terpenoid and phenylpropanoid metabolism in lettuce is also interconnected, which is consistent with previous studies demonstrating a similar metabolic link in other plant species, such as *Artemisia annua* [89] and sweet basil (*Ocimum basilicum*) [19]. Benzaldehyde is important for the plant’s life cycle—for example, by attracting pollinators [90,91], and in medical research due to its anticancer properties [92]. It is also used as a flavoring agent in both food and pharmaceutical products due to its characteristic almond- and cherry-like aroma [18,93]. The marked increase in benzaldehyde in *Lshmgr7* may improve the flavor of lettuce and, hence, its palatability and consumer appeal. Moreover, enhanced aroma can mask the perception of lettuce leaves’ inherent bitterness [94,95,96]. In the future, integrating the Lshmgr7 modification—which enhances flux into terpenoid and phenylpropanoid pathways—into nutrient-enriched, gene-edited lettuce lines high in antioxidants, provitamins, and dietary fibers [33,35] could allow for the creation of a ‘hyper-charged’ lettuce with high nutritional value.

## 3. Materials and Methods

### 3.1. Plant Material and Growth Conditions

*Petunia × hybrida* cv. Mitchell diploid plants were grown from seeds. Plants were grown in a greenhouse under 25 °C/20 °C day/night temperatures with a 16 h light/8 h dark photoperiod (incandescent light bulbs, 120 V, 16 lm/W, were used to provide additional lighting). Romaine lettuce cv. Noga seeds were kindly provided by Hazera Ltd. (Brurim, Israel). The plants were grown in a greenhouse under 26 °C/20 °C day/night temperatures and natural lighting. Light intensity in the greenhouses was continuously monitored throughout the growth period using a HOBO Pendant Temperature Data Logger (MX2201, Onset Brands, Bourne, MA, USA; https://www.onsetcomp.com/).

### 3.2. Phylogenetic Analysis

Protein sequences were aligned using the Clustal W algorithm [97] implemented in MEGA11 [98] with default parameters (gap opening penalty 10.00; gap extension penalty 0.20). Maximum likelihood with default parameters (1000 bootstraps; Jones–Taylor–Thornton substitution model) was used as the statistical method for phylogenetic analysis in MEGA11.

### 3.3. Vector Construction

Tobacco rattle virus RNA2 (pTRV2) vectors pTRV2-sgRNA1-sgRNA2 and pTRV2-sgRNA1-sgRNA3 for petunia, and pTRV2-Ls-sgRNA1-Ls-sgRNA2 for lettuce, were synthesized by Twist Bioscience (http://www.twistbioscience.com/), based on the pTRV2 vector with GenBank accession AF406991 [99].

### 3.4. Generation of Petunia Phhmgr1 and Lettuce Lshmgr7 Gene-Edited Lines

*Agrobacterium tumefaciens* (strain AGL0) cells carrying tobacco rattle virus RNA1 (pTRV1) (AF406990 [99]) or the pTRV2 vectors were incubated overnight in the dark at 28 °C and grown in LB Broth Lennox medium (Formedium, Norfolk, UK) supplemented with 100 μM acetosyringone (Sigma, St. Louis, MO, USA) and 50 μg/mL kanamycin. Bacterial cells were centrifuged at 6000× *g* for 5 min at room temperature, washed, and resuspended with inoculation solution containing 100 μM acetosyringone (for petunia) or 100 μM acetosyringone with 10 mM MgCl_2_ (for lettuce). Agrobacteria carrying pTRV1 and either of the pTRV2 vectors were mixed at a 1:1 ratio (to OD_600_ = 0.5).

#### 3.4.1. Petunia

Sterile explants (young leaves) from *Cas9*-expressing petunia cv. Mitchell [34] were incubated for 20 min in the *Agrobacterium* solutions (in LB) and then incubated in the dark for 3 days on Murashige and Skoog (MS) medium supplemented with 3% (*w*/*v*) sucrose, followed by transfer for selection on MS with 3% sucrose, antibiotics (kanamycin, carbenicillin), and phytohormones (1.5 mg/L 6-benzyladenine [BA] and 0.15 mg/L 1-naphthaleneacetic acid [NAA]), and incubated under a light/dark 12 h/12 h photoperiod at 22 °C until regeneration and elongation of regenerants were observed. The medium was renewed every 2 weeks. At the elongation stage, the BA concentration was decreased to 1 mg/L. To switch regenerants to rooting, they were transferred to medium without BA supplemented with 0.25 mg/L NAA. Regenerated plantlets were tested for the presence of mutations by PCR (primers are shown in Table S1) followed by Sanger sequencing. Edited plants were further grown and self-pollinated to obtain homozygous mutated lines. T2 progeny were used for the experiments.

#### 3.4.2. Lettuce

Sterile cotyledons of 6-day-old seedlings from *Cas9*-expressing lettuce cv. Noga [35] were dissected to include the petiole and immersed in *Agrobacterium* solution for 10 min at room temperature. Inoculated cotyledons were placed between two filter papers on a co-cultivation medium (1× MS, 3% sucrose, 100 μM acetosyringone) and incubated in the dark for 2 days. *DsRed*-expressing cotyledons were then transferred to regeneration medium (1× MS, 3% sucrose, 300 mg/L carbenicillin, 0.5 mg/L BA, 0.04 mg/L NAA). Cotyledons were subcultured onto fresh medium every 5–7 days until distinct plantlets developed. Regenerated plantlets were tested for the presence of mutations by PCR (primers are shown in Table S1) followed by Sanger sequencing, and edited plantlets were transferred to rooting medium (1× MS, 3% sucrose). Edited plants were further grown and self-pollinated to obtain homozygous mutated lines. T2 progeny were used for the experiments.

### 3.5. Collection of Emitted Volatiles and Internal Pools

Dynamic headspace analysis was performed on petunia flowers by harvesting flowers 1DPA and placing them in a 50 mL beaker filled with tap water in jars (2 flowers per jar). For lettuce, 5 g of fully expanded middle leaves from 2-month-old lettuce plants were ground in liquid nitrogen using a mortar and pestle. The resulting powder was transferred into a 50 mL glass beaker, which was then placed in a jar. Volatiles were collected for 24 h using columns connected to a vacuum pump at a flow rate of 2 L/min. Volatiles were collected for 24 h using columns made of glass tubes containing 100 mg Porapak Q polymer and 100 mg 20/40-mesh, held in place with plugs of silanized glass wool. Localized headspace collection was performed as described in Skaliter et al. [45]. Briefly, petunia flowers were harvested 1DPA and immediately placed in 50 mL beakers filled with tap water (1 flower per beaker). A collection column (see above), fitted with a pipette tip that was cut at the top, was carefully placed over the same petal section of each flower. Volatiles were collected for 24 h using columns connected to a vacuum pump at a flow rate of 0.5 L/min. Trapped volatiles were eluted with 1.5 mL hexane and 0.5 mL acetone. Isobutylbenzene was used as an internal standard.

To determine the pool sizes of volatile compounds, 100 mg tissue from 1DPA flowers was ground in liquid nitrogen and extracted in 400 μL hexane containing isobutylbenzene as the internal standard. Following 2 h of incubation with gentle shaking at 25 °C, extracts were centrifuged at 10,500× *g* for 10 min, and the supernatant was further centrifuged, followed by GC–MS analysis.

### 3.6. GC–MS Analysis

GC–MS analysis (of a 1 μL sample) was performed using a device consisting of a PAL autosampler (CTC Analytics, Zwingen, Switzerland), a TRACE GC 2000 gas chromatograph (Thermo Fisher Scientific, Waltham, MA, USA) equipped with an Rtx-5SIL mass spectrometer fused-silica capillary column (inner diameter 0.25 μm, 30 m × 0.25 mm; Restek, Centre County, PA, USA), and a TRACE DSQ quadruple mass spectrometer (Thermo Fisher Scientific, Waltham, MA, USA). Helium was used as the carrier gas at a flow rate of 1 mL/min. The injector temperature was set to 220 °C (splitless mode), the interface to 240 °C, and the ion source to 200 °C. The analysis was performed under the following temperature program: 2 min of isothermal heating at 40 °C followed by a 7 °C/min oven temperature ramp to 250 °C, then 2 min of isothermal heating. The system was equilibrated for 1 min at 70 °C before injecting the next sample. Mass spectra were recorded at 3.15 scans/s with a scanning range of 40–350 mass-to-charge ratio and electron energy of 70 eV. Compounds were tentatively identified (>95% match) based on NIST/EPA/NIH Mass Spectral Library data version NIST 17 (with software version 3.4) using xcalibur 1.3 (Thermo Fisher Scientific, Waltham, MA, USA). Further identification of all phenylpropanoid compounds and β-ionone was based on a comparison of mass spectra and retention times with those of authentic standards (Sigma-Aldrich, St. Louis, MO, USA) analyzed under similar conditions.

### 3.7. RNA Extraction and RT-qPCR Analyses

Total RNA was extracted from 30–100 mg of ground (with liquid nitrogen) floral tissues using the Tri-Reagent kit (Sigma-Aldrich) and treated with RNase-free DNase I (Thermo Fisher Scientific). First-strand cDNA was synthesized using total RNA, oligo(dT) primer, and reverse transcriptase ImProm-II (Promega, Madison, WI, USA) according to the manufacturer’s instructions. Two-step real-time qPCR was performed on a CFX Opus 384 Real-Time PCR System (Bio-Rad, Hercules, CA, USA) using 2X qPCRBIO SyGreen Blue Mix Hi-ROX (PCR Biosystems, London, UK). A standard curve was generated for each gene using dilutions of cDNA samples, and data analysis was performed using Bio-Rad CFX Maestro software (Bio-Rad, Hercules, CA, USA). Primer specificity was determined by melting-curve analysis. Raw transcript-level data were normalized to the geomean of *EF1α* and *Ubiquitin*. Quantification calculations were carried out using the 2^−ΔΔCT^ formula. Primers are shown in Table S1.

### 3.8. Statistical Analysis

Statistical analyses were performed using JMP Pro 18 (SAS, Cary, NC, USA).

## 4. Conclusions

This work demonstrates the successful implementation of a virus-based CRISPR/Cas9 system to modify specific sites within a target gene—*HMGR*—in petunia and lettuce. While knockout of *HMGR* led to deleterious effects, specific mutations in the N-terminus region led to enhancement of various metabolites from distinct pathways in both plant species, including sesquiterpenes, apocarotenoids, and C6-C1/C2/C3 phenylpropanoids. Gene-expression analysis suggested that the HMGR modification increased carbon flux into these pathways, resulting in enhanced production of these metabolites. The finding that several metabolic pathways were affected by the *HMGR* modification allowed us to reveal complex layers of interaction between the MVA, MEP, and phenylpropanoid pathways, shedding light on the intricate network of specialized metabolism in plants. Moreover, given the importance of terpenoids and phenylpropanoids for humans in medicine, flavor, fragrance, and pigmentation, this research may contribute to the development of value-added ornamental and food crops.

## Data Availability

The original contributions presented in this study are included in the article/Supplementary Material. Further inquiries can be directed to the corresponding author.

## Supplementary Materials

The following supporting information can be downloaded at: https://www.mdpi.com/article/10.3390/ijms27031522/s1.
