# Supplementary material for: Targeted Gene Modification of HMGR Enhances Biosynthesis of Terpenoid and Phenylpropanoid Volatiles in Petunia and Lettuce
Source: Int J Mol Sci. 2026 Feb 4;27(3):1522. doi: 10.3390/ijms27031522 (PMC12898840; doi:10.3390/ijms27031522)
Supplement: Supplementary file 1 [file ijms-27-01522-s001.zip › ijms-4119537 Supplementary Figures.pdf]

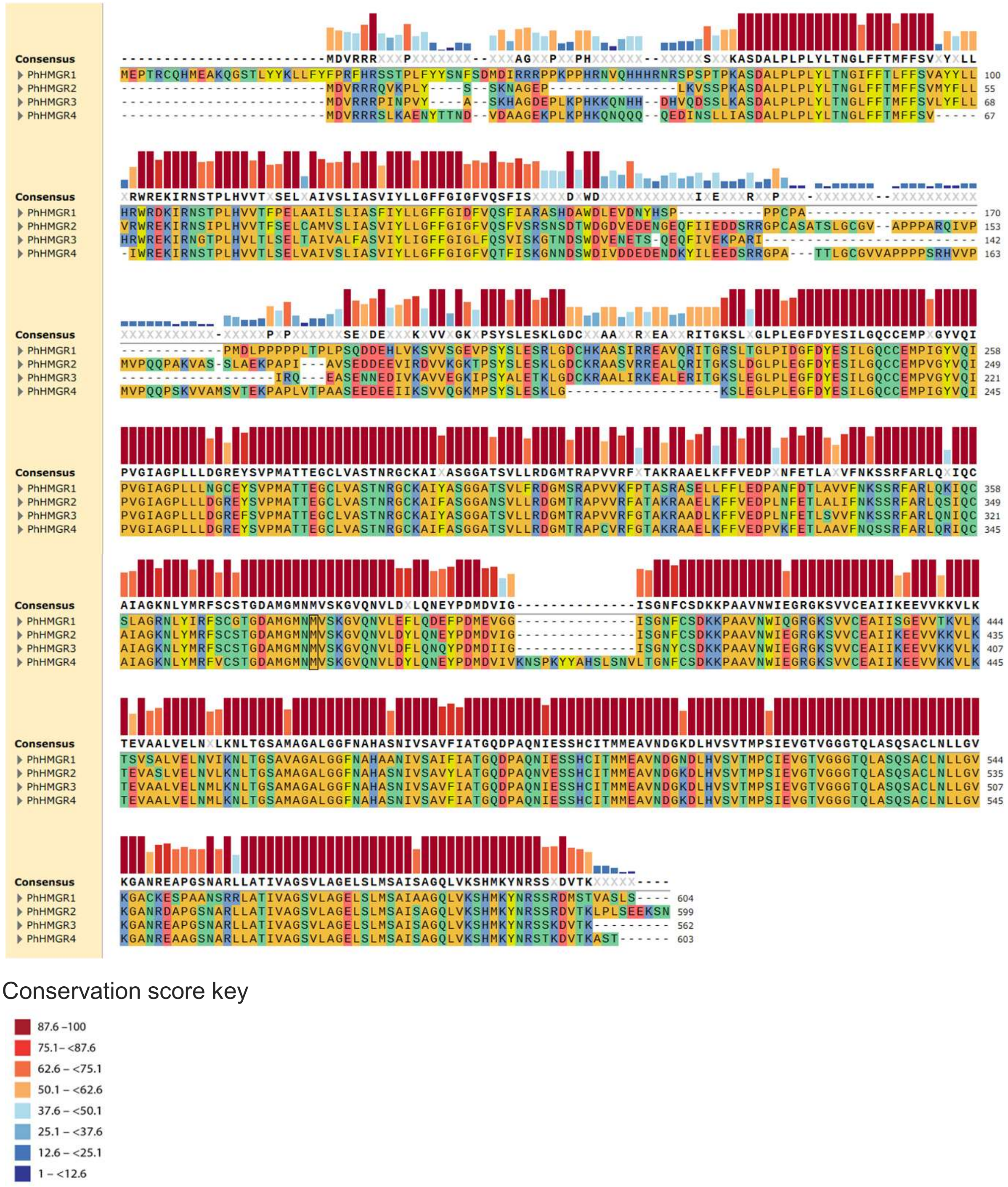

Figure S1. Protein sequence alignment of *Petunia* × *hybrida* 3-hydroxy-3-methylglutaryl-coenzyme A reductases (PhHMGRs). Clustal Omega alignment was conducted using SnapGene software (version 8.2.2). Conservation is indicated by bars (see key) above the alignment, and residues with >50% consensus are shown below the bars. Amino acids are colored according to their physicochemical properties.

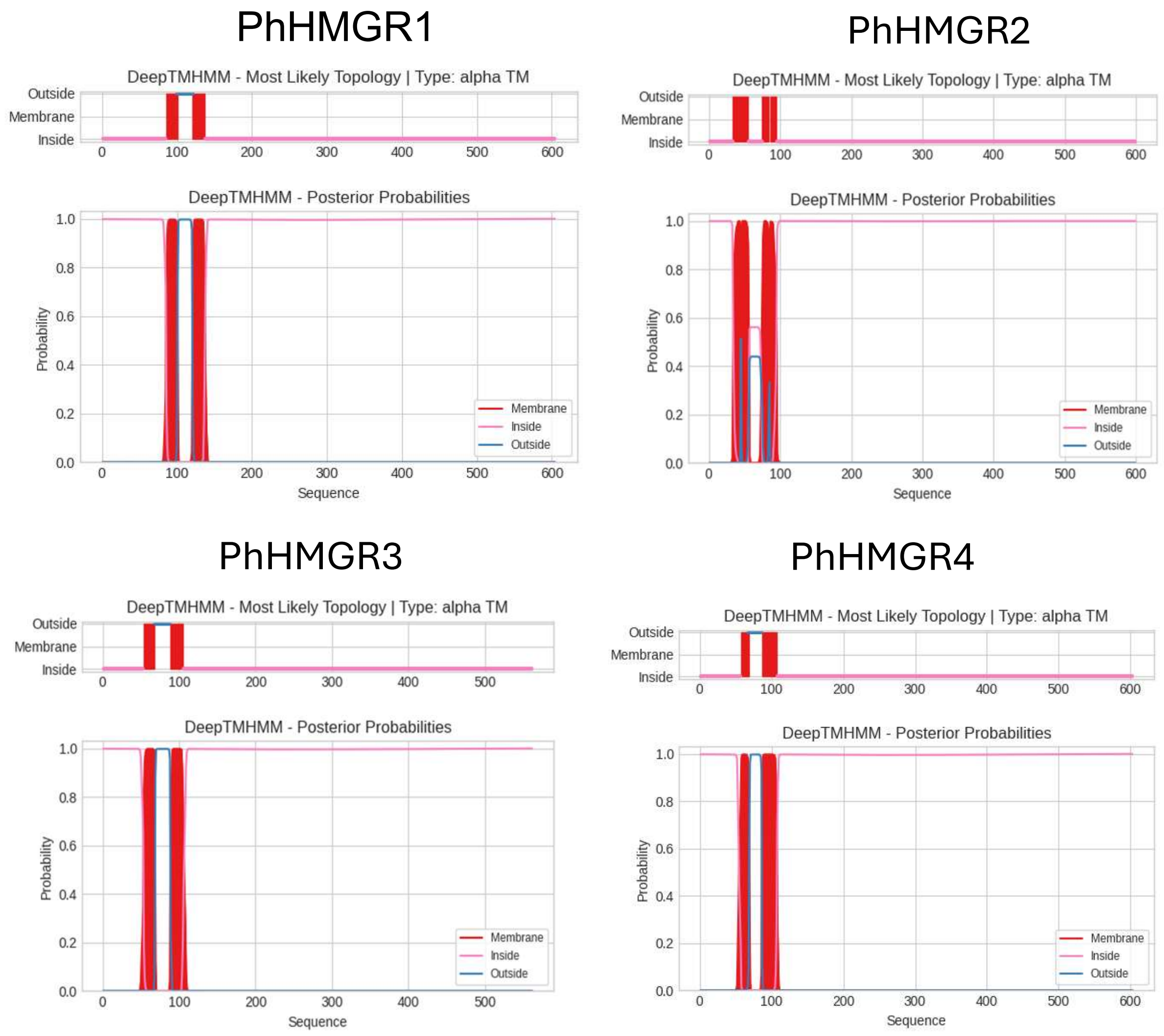

Figure S2. Prediction of the membrane topology of HMGRs in petunia. All PhHMGR proteins are predicted to contain two transmembrane domains. The analysis was performed using DeepTMHMM (https://dtu.biolib.com/DeepTMHMM).

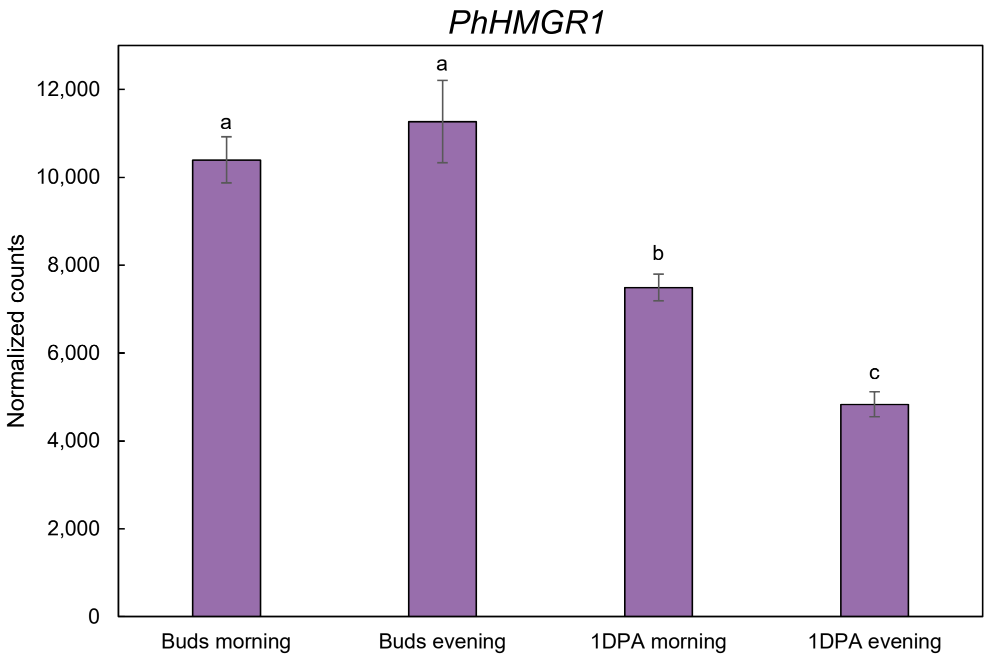

Figure S3. Developmental and diurnal expression pattern of *PhHMGR1*. Expression levels were analyzed in petunia floral buds and flowers 1 day postanthesis (1DPA) in the morning (1000 h) and evening (1900 h). Transcripts were detected by RNA-sequencing (Shor *et al*., 2023) [43]. Data are means ± SEM (n = 3). Significance of differences was calculated by Tukey's multiple comparison test following one-way ANOVA. Values with different letters are significantly different at $p \leq 0.05$. Standard errors are indicated by vertical lines.

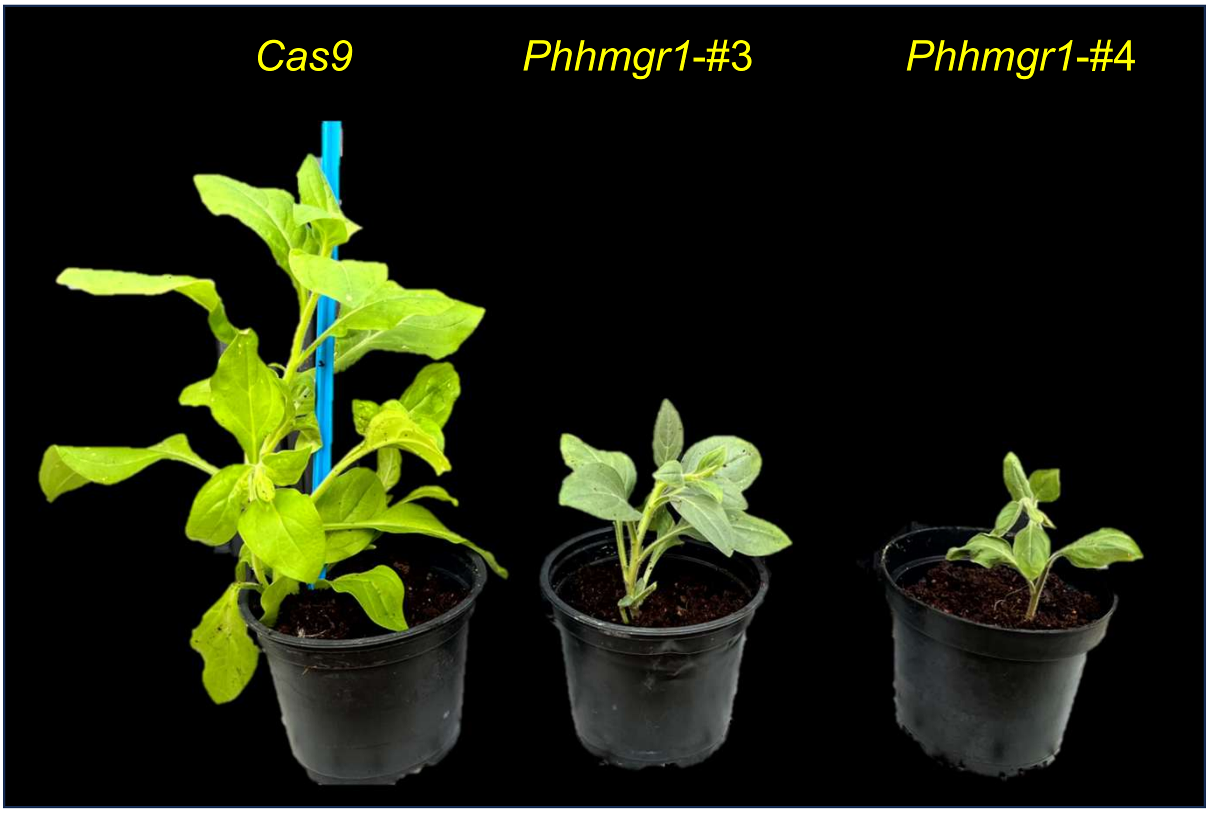

Figure S4. Frameshift mutations in *Phhmgr1* result in dwarfed plants. Representative 1-month-old plants of *Cas9* control and *Phhmgr1*-#3 and #4.

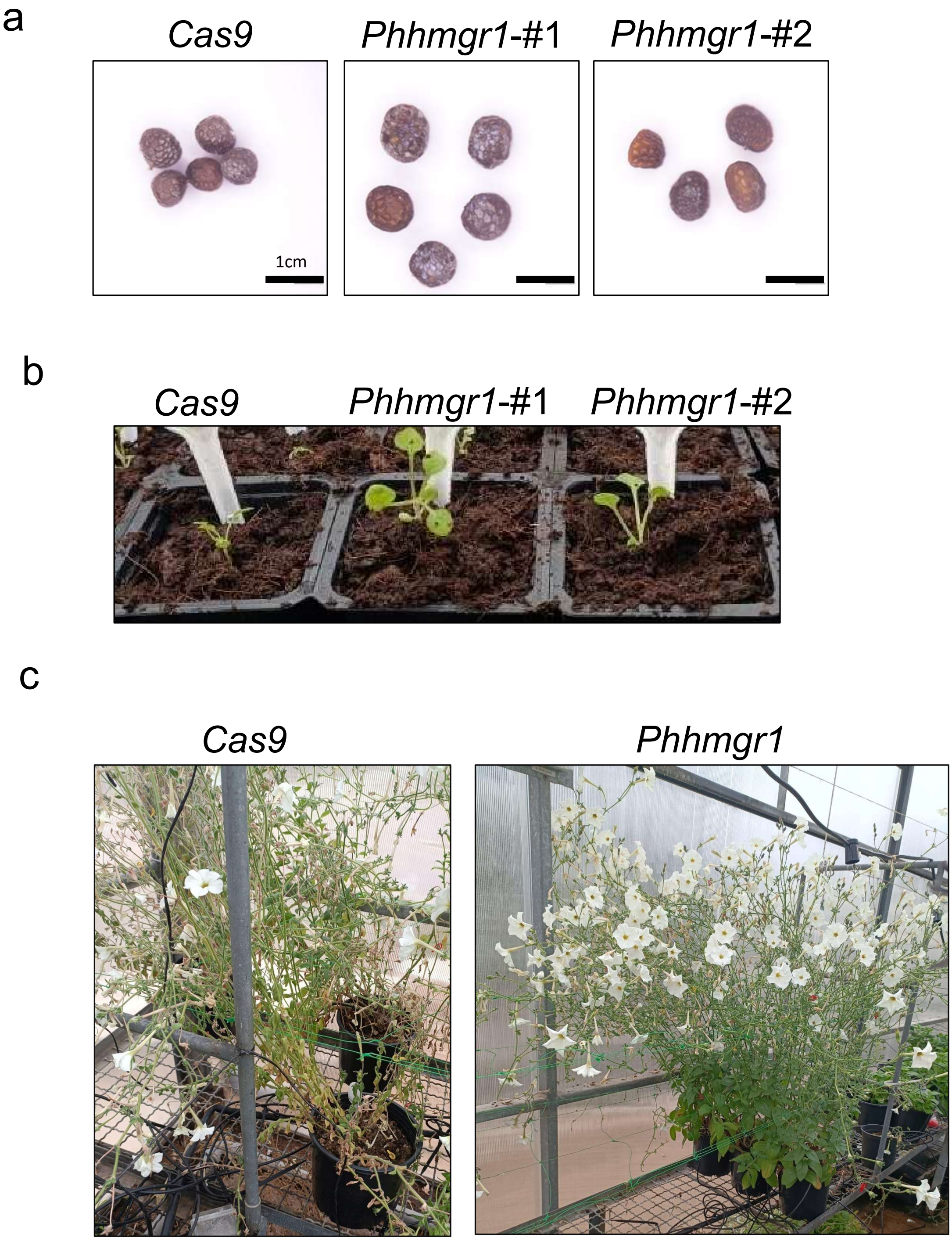

Figure S5. Gene-editing of *PhHMGR1* results in vigorous plants with bigger seeds. (**a**) 12-day-old seedlings, (**b**) 6-month-old plants, and (**c**) flowering plants of *Cas9* control and *Phhmgr1* lines.

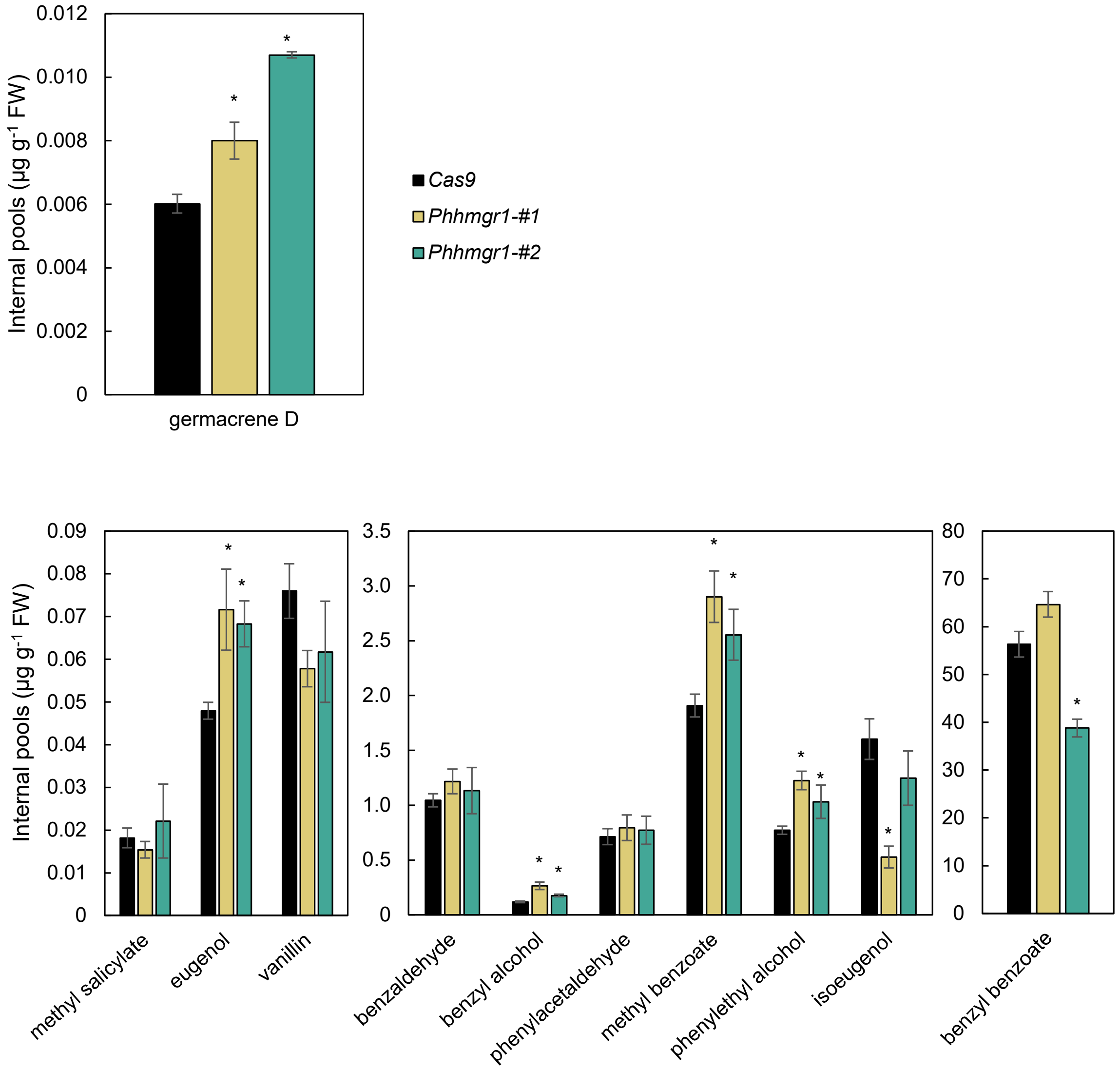

Figure S6. Gene-editing of *PhHMGR1* leads to altered accumulation of terpenoids and phenylpropanoids in petunia flowers. Internal pools were extracted from flowers of control *Cas9* and *Phhmgr1-#1* and *#2* at 1 day postanthesis followed by GC–MS analysis. Data are means ± SEM (n = 4–10). Significance of differences was calculated using Dunnett's or Steel's test (*$p \leq 0.05$) with *Cas9* as the control following one-way ANOVA. Standard errors are indicated by vertical lines.

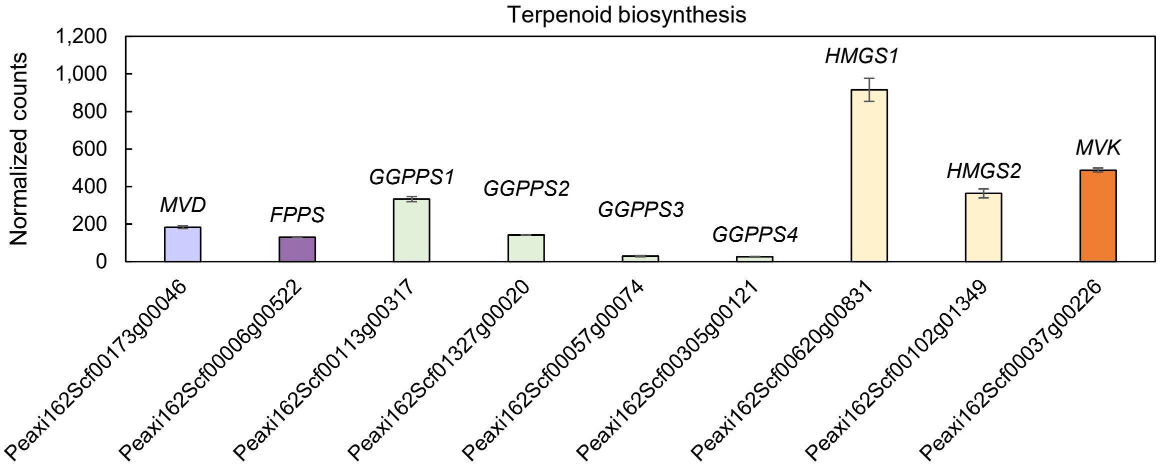
Terpenoid biosynthesis
Normalized counts
1,200
1,000
800
600
400
200
0
MVD
FPPS
GGPPS1
GGPPS2
GGPPS3
GGPPS4
HMGS1
HMGS2
MVK
Peaxi162Scf00173g00046
Peaxi162Scf00006g00522
Peaxi162Scf00113g00317
Peaxi162Scf01327g00020
Peaxi162Scf00057g00074
Peaxi162Scf00305g00121
Peaxi162Scf00620g00831
Peaxi162Scf00102g01349
Peaxi162Scf00037g00226

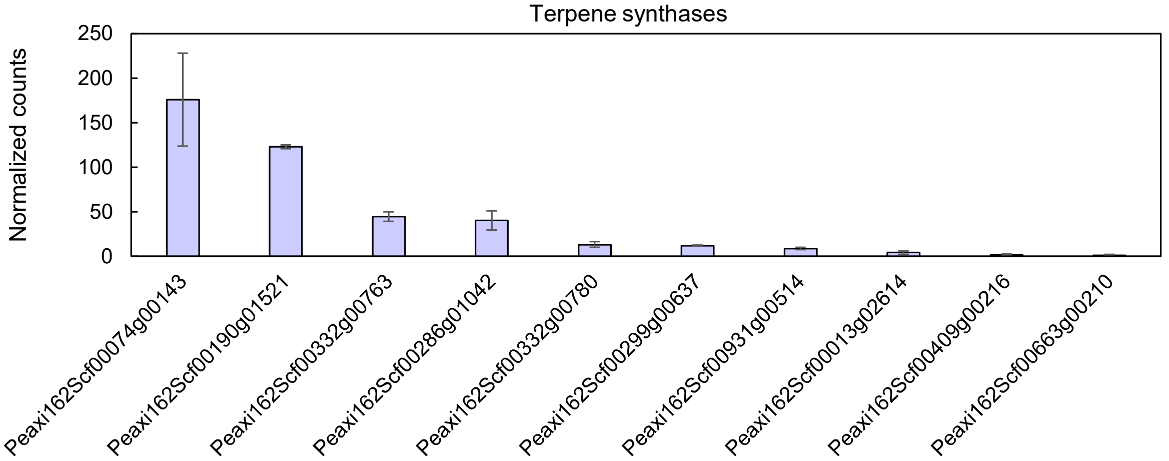
Terpene synthases
Normalized counts
250
200
150
100
50
0
Peaxi162Scf00074g00143
Peaxi162Scf00190g01521
Peaxi162Scf00332g00763
Peaxi162Scf00286g01042
Peaxi162Scf00332g00780
Peaxi162Scf00299g00637
Peaxi162Scf00931g00514
Peaxi162Scf00013g02614
Peaxi162Scf00409g00216
Peaxi162Scf00663g00210

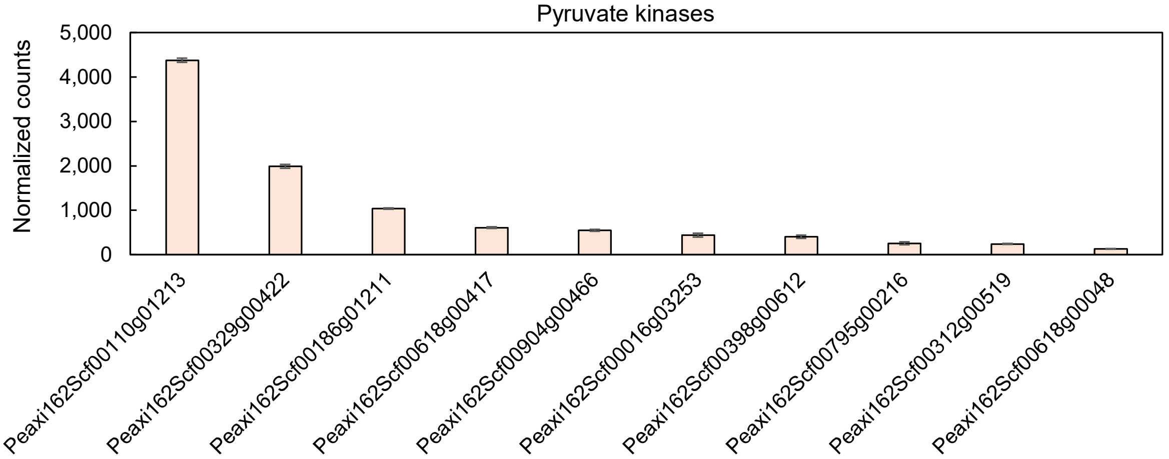
Pyruvate kinases
Normalized counts
5,000
4,000
3,000
2,000
1,000
0
Peaxi162Scf00110g01213
Peaxi162Scf00329g00422
Peaxi162Scf00186g01211
Peaxi162Scf00618g00417
Peaxi162Scf00904g00466
Peaxi162Scf00016g03253
Peaxi162Scf00398g00612
Peaxi162Scf00795g00216
Peaxi162Scf00312g00519
Peaxi162Scf00618g00048

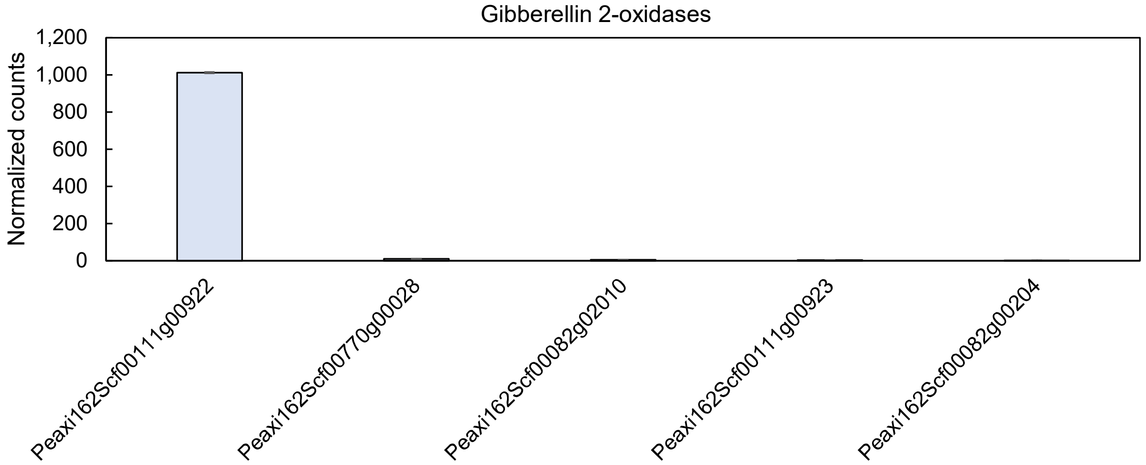
Gibberellin 2-oxidases
Normalized counts
1,200
1,000
800
600
400
200
0
Peaxi162Scf00111g00922
Peaxi162Scf00770g00028
Peaxi162Scf00082g02010
Peaxi162Scf00111g00923
Peaxi162Scf00082g00204

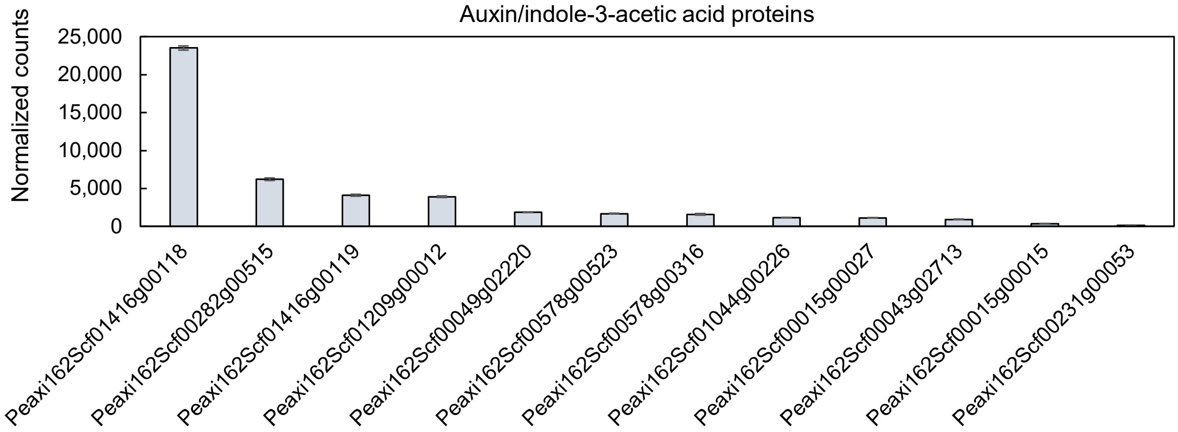

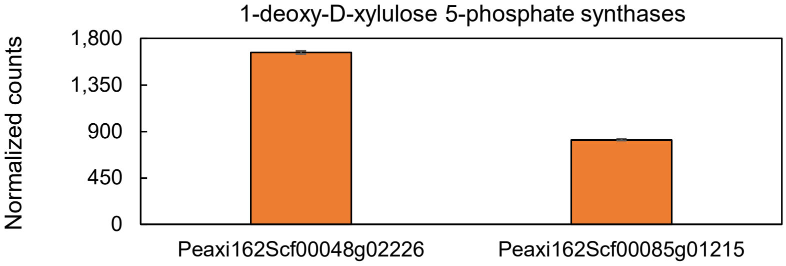

Figure S7. Expression levels of putative genes encoding factors involved in terpenoid biosynthesis, pyruvate metabolism, and hormone biosynthesis/signaling in petunia petals. Expression levels were analyzed in petunia flowers 1DPA. Transcripts were obtained from the petunia floral transcriptome (Shor *et al.*, 2023) [43]. Data are means ± SEM (*n* = 3). Standard errors are indicated by vertical lines. Enzyme abbreviations: FPP synthase, FPPS; GGPP synthase 1–4, GGPPS1–4; HMG-CoA synthase 1/2, HMGS1/2; mevalonate 5-diphosphate decarboxylase, MDD; mevalonate kinase, MVK.

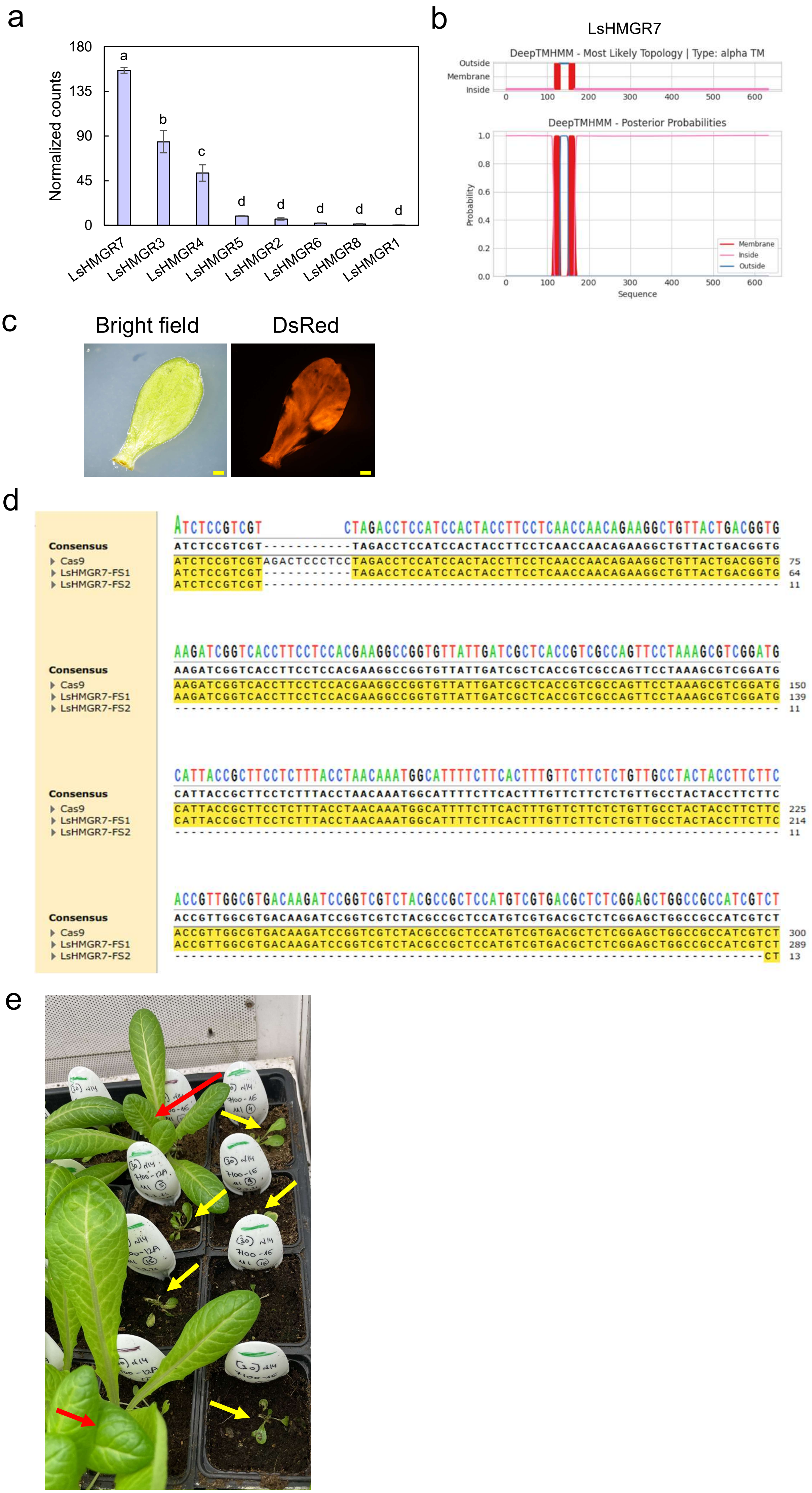
a
Normalized counts
180
135
90
45
0
a
b
c
d
LsHMGR7
LsHMGR3
LsHMGR4
LsHMGR5
LsHMGR2
LsHMGR6
LsHMGR8
LsHMGR1
b
LsHMGR7
DeepTMHMM - Most Likely Topology | Type: alpha TM
Outside
Membrane
Inside
DeepTMHMM - Posterior Probabilities
Probability
Sequence
Membrane
Inside
Outside
c
Bright field
DsRed
d
Consensus
Cas9
LsHMGR7-FS1
LsHMGR7-FS2
ATCTCCGTCGT TAGACCTCCATCCACTACCTTCCTCAACCAACAGAAGGCTGTTACTGACGGTG
AAGATCGGTCACCTTCCTCCACGAAGGCCGGTGTTATTGATCGCTCACCGTCGCCAGTTCCTAAAGCGTCGGATG
CATTACCGCTTCCTCTTTACCTAACAAATGGCATTTTCTTCACTTTGTTCTTCTCTGTTGCCTACTACCTTCTTC
ACCGTTGGCGTGACAAGATCCGGTCGTCTACGCCGCTCCATGTCGTGACGCTCTCGGAGCTGGCCGCCATCGTCT
75
64
11
150
139
11
225
214
11
300
289
13
e

Figure S8. Generation of lettuce gene-edited lines. (**a**) Expression levels of *LsHMGR*s in above-ground tissues. Transcripts levels were obtained from transcriptome analysis performed in Sunchung *et al.* (2020) [80]. Data are means ± SEM ($n = 3$). Significance of differences was calculated by Tukey's multiple comparison test following one-way ANOVA. Values with different letters are significantly different at $p \leq 0.05$. Standard errors are indicated by vertical lines; (**b**) Prediction of the membrane topology of LsHMGR7 using DeepTMHMM (https://dtu.biolib.com/ DeepTMHMM) showing that it has two transmembrane domains; (**c**) Representative cotyledons of lettuce expressing DsRed. Cotyledon explants were inoculated with the pTRV2 construct harboring Ls-sgRNA1, sgRNA2 and DsRed marker gene, serving as a reporter for TRV expression. Explants were examined under a fluorescent stereomicroscope (SMZ1270, Nikon, https://www.nikon.com/). The images were taken 7 days postinoculation. Bar = 1 mm; (**d**) Targeted genomic sequences of *Cas9* control and *LsHMGR7*-edited lines carrying frameshift (FS) mutations: *LsHMGR7-FS1* and *LsHMGR7-FS2*, harboring 11- and 287-bp deletions, respectively. Clustal Omega alignment was performed using SnapGene software (version 8.2.2); (**e**) Representative 1-month-old *Cas9* control plantlets (red arrows) and dwarfed plantlets with frameshift mutations (yellow arrows).
